# Anti-CD19-engineered exosomes enable B cell-targeted anti-BAFF mRNA delivery to alleviate lupus progression

**DOI:** 10.1016/j.ymthe.2026.08.040

**Published:** 2026-09-29

**Authors:** Linda Zeng, Jingxian Shu, Shuping Zhong, Xin Dong, Yongjian Wu, Xi Huang

## Main text

(Molecular Therapy *34*, 3417–3435; June 2026)

In the originally published version of this article, the authors identified an inadvertent error in the drug name labeling that requires correction to ensure accuracy and clarity for readers; specifically, “Tabalumab” was used in the original publication but has now been corrected to “Sandy-2.” The article has been updated to reflect this. This error arose from proofreading oversight during final preparation and does not affect any experimental results, data interpretation, or the main conclusions of the study. All authors confirm the correction and take full responsibility for this oversight.

