# Supplementary material for: Anti-CD19-engineered exosomes enable B cell-targeted anti-BAFF mRNA delivery to alleviate lupus progression
Source: Mol Ther. 2026 Feb 24;34(6):3417–35. doi: 10.1016/j.ymthe.2026.01.037 (PMC13238940; doi:10.1016/j.ymthe.2026.01.037)
Supplement: Supplementary file 1 [file mmc1.pdf]

YMTHE, Volume *34*

Supplemental Information

Anti-CD19-engineered exosomes enable B cell-targeted anti-BAFF mRNA delivery to alleviate lupus progression

Linda Zeng, Jingxian Shu, Shuping Zhong, Xin Dong, Yongjian Wu, and Xi Huang

Table S1 Sequences of primers

Name of primers	Sequences of primers
Mouse-β-actin	Forward: GATTACTGCTCTGGCTCCTAGC<br>Reverse: GACTCATCGTACTCCTGCTTGC
Mouse-GAPDH	Forward: GGAGAGTGTTTCCTCGTCCC<br>Reverse: ATGAAGGGGTCGTTGATGGC
Mouse-IL-4	Forward: ATCATCGGCATTTTGAACGAGGTC<br>Reverse: ACCTTGGAAGCCCTACAGACGA
Mouse-IL-10	Forward: CGGGAAGACAATAACTGCACCC<br>Reverse: CGGTTAGCAGTATGTTGTCCAGC
Mouse-TNF-α	Forward: GGTGCCTATGTCTCAGCCTCTT<br>Reverse: GCCATAGAACTGATGAGAGGGAG
Mouse-IL-1β	Forward: TGGACCTTCCAGGATGAGGACA<br>Reverse: GTTCATCTCGGAGCCTGTAGTG
Mouse-TGF-β	Forward: TGATACGCCTGAGTGGCTGTCT<br>Reverse: CACAAGAGCAGTGAGCGCTGAA
Mouse-IL-6	Forward: TACCACTTCACAAGTCGGAGGC<br>Reverse: CTGCAAGTGCATCATCGTTGTTC
Mouse-IL-12p40	Forward: TCACGCTACCTCCTCTTT<br>Reverse: GGTTTCGGGACTGGCTAA

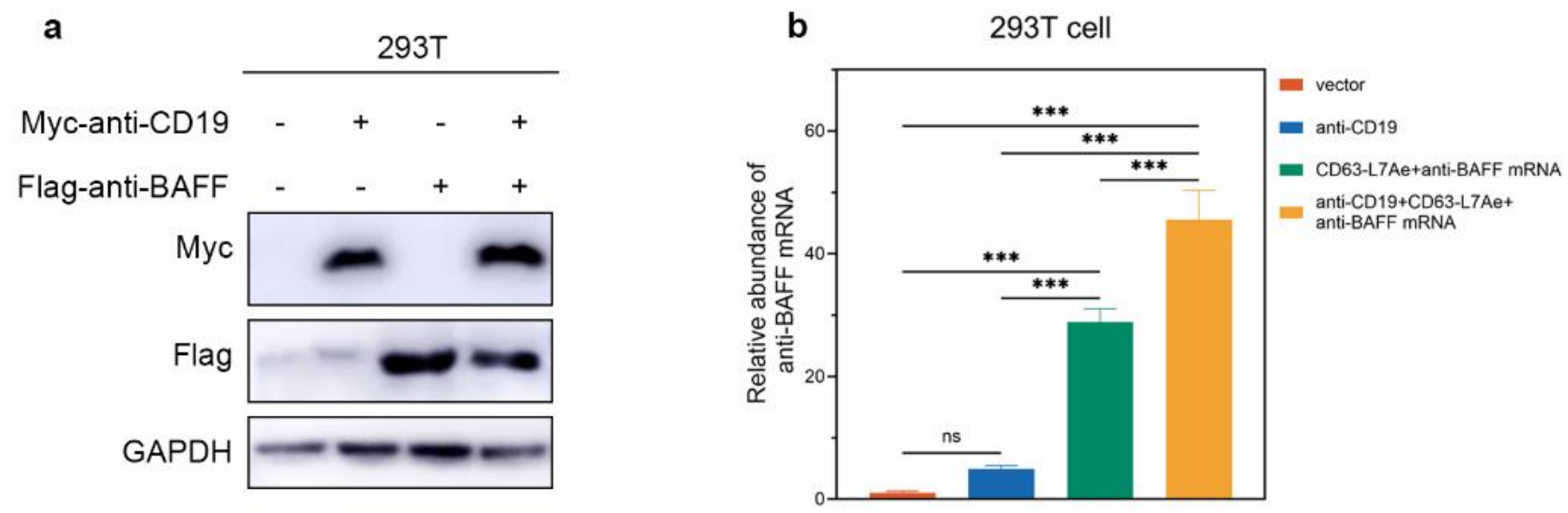

Figure S1 Characterization of stable transfected HEK293T cell line.

(a) The expression of Myc-anti-CD19 and Flag-anti-BAFF in each stable cell line were analyzed by Western blotting. **(b)** The relative abundance of anti-BAFF mRNA in each stable cell line was analyzed by quantitative real-time PCR. Data are presented as the mean±SEM of three biological replicates. **p* < 0.05, ***p* < 0.01, ****p* < 0.005, ns: not significant.

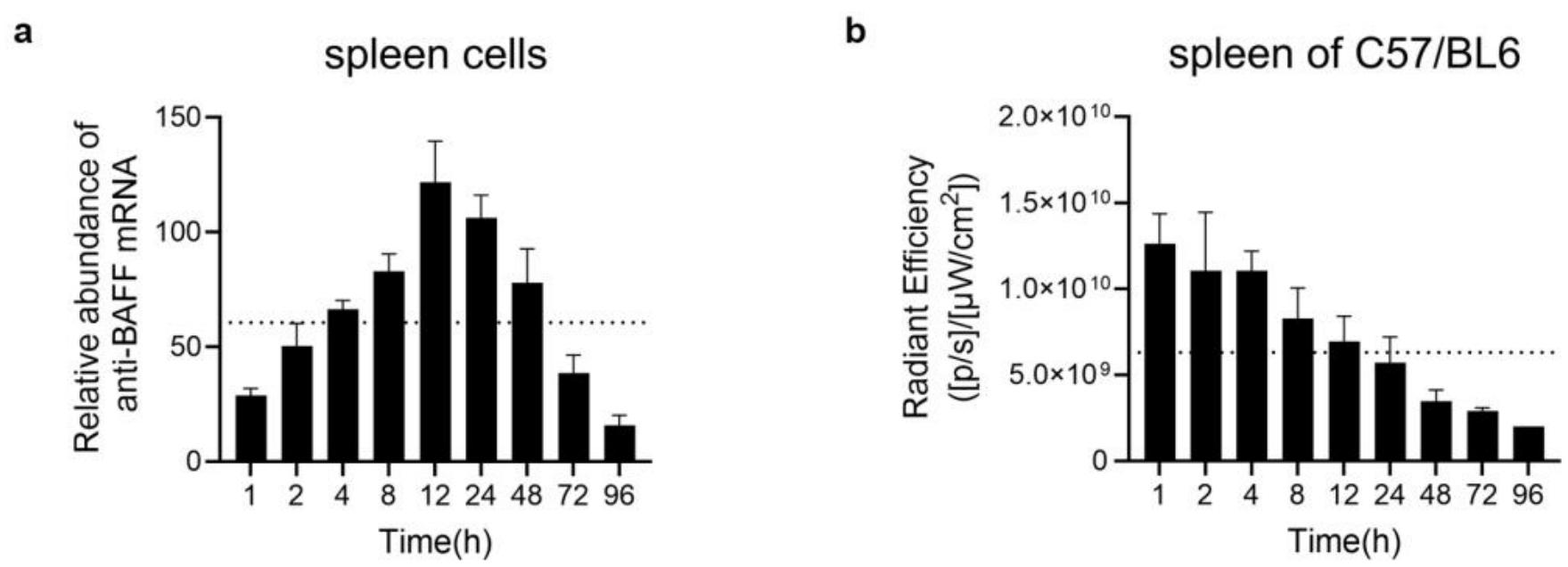

Figure S2 *In vivo* half-life of CD19@anti-BAFF^{mRNA} Exo.

(a) The relative abundance of anti-BAFF mRNA in the spleen was quantified by qPCR at 1, 2, 4, 8, 12, 24, 48, 72, 96 hours after the injection of CD19@anti-BAFF^{mRNA} Exo. The dashed line corresponds to 50% of the detected peak value for the relative abundance of mRNA levels. (b) The radiant efficiency of CD19@anti-BAFF^{mRNA} Exo (stained with DiR) in the spleen was statistically analyzed at 1, 2, 4, 8, 12, 24, 48, 72, 96 hours after injection. The dashed line corresponds to 50% of the detected peak value for the relative radiant efficiency.

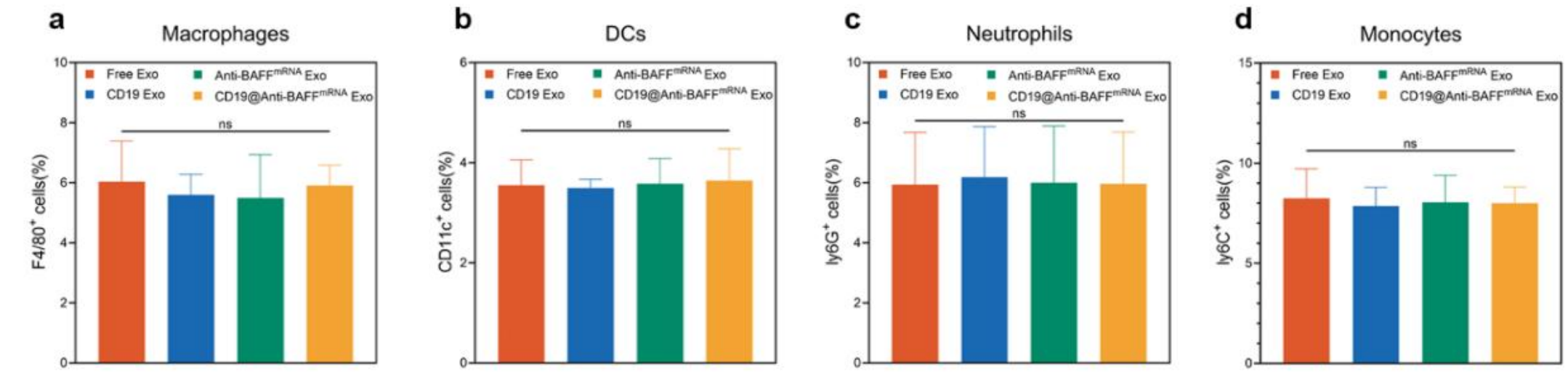

Figure S3 Proportions of immune cells in splenic cells of ALD-DNA induced lupus mice treated by different groups of exosomes.

(a) Proportions of macrophages, **(b)** DCs, **(c)** neutrophils and **(d)** monocytes in splenic cells of ALD-DNA induced lupus mice treated by different groups of exosomes. Data are presented as the mean±SEM of three biological replicates. ns: not significant.

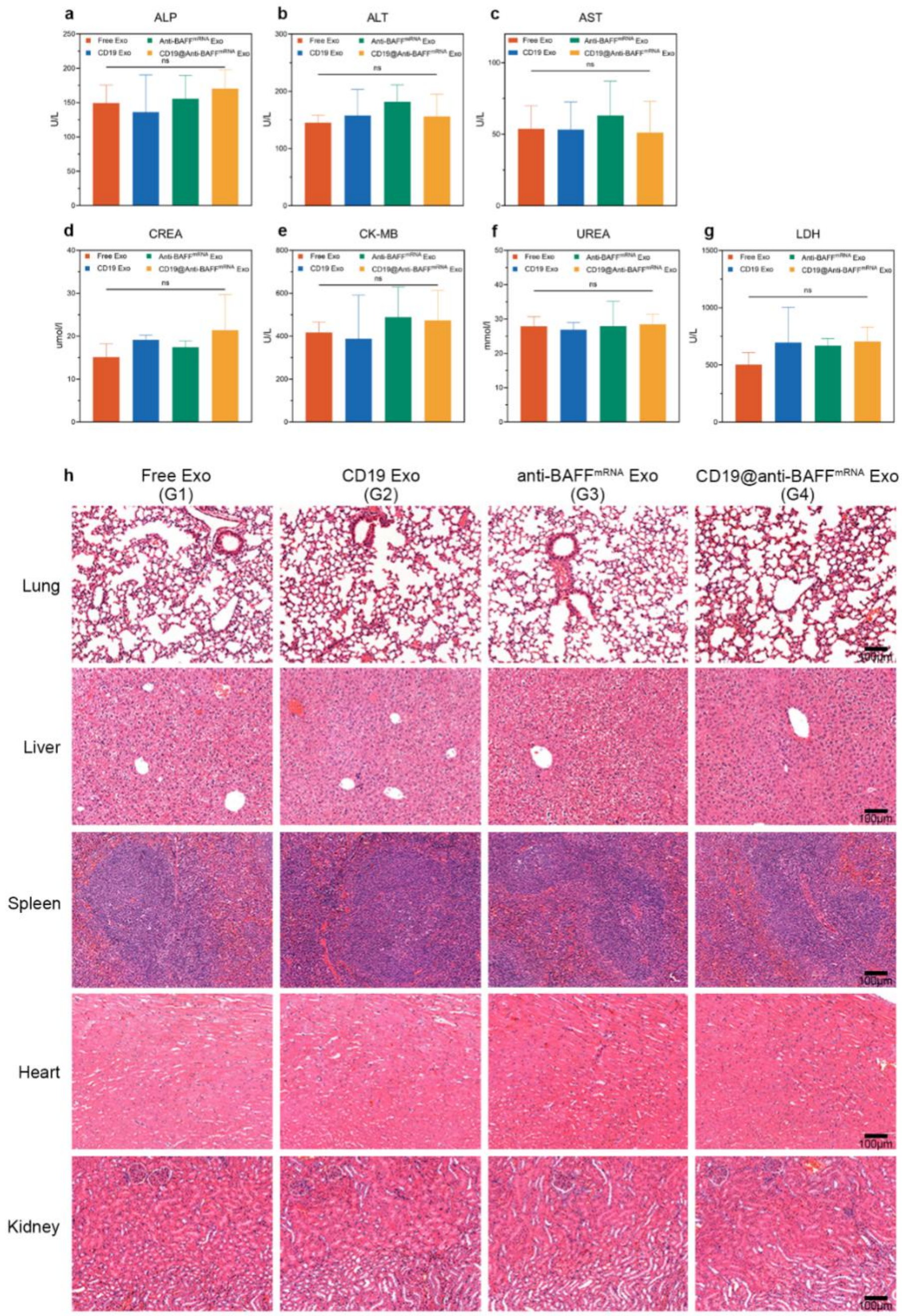

Figure S4 Biocompatibility of CD19@anti-BAFF^{mRNA} exosomes.

(a-g) The levels of biochemical indicators (ALP, ALT, AST, CREA, CK-MB, UREA and LDH) of wild-type mice treated with different groups of exosomes. **(h)** Representative images of H&E-stained slices of major organs (lung, liver, spleen, heart and kidney) of wild-type mice treated with different groups of exosomes.
