# Supplementary material for: Anti-CD19-engineered exosomes enable B cell-targeted anti-BAFF mRNA delivery to alleviate lupus progression
Source: Mol Ther. 2026 Feb 24;34(6):3417–35. doi: 10.1016/j.ymthe.2026.01.037 (PMC13238940; doi:10.1016/j.ymthe.2026.01.037)
Supplement: Supplementary file 2 [file mmc2.pdf]

Molecular Therapy
Original Article

Anti-CD19-engineered exosomes enable B cell-targeted anti-BAFF mRNA delivery to alleviate lupus progression

Linda Zeng,[1,4] Jingxian Shu,[1,2,4] Shuping Zhong,[3] Xin Dong,[1] Yongjian Wu,[1] and Xi Huang[1]

[1]Center for Infection and Immunity and Guangdong Provincial Engineering Research Center of Molecular Imaging, The Fifth Affiliated Hospital of Sun Yat-sen University, Zhuhai, Guangdong Province 519000, China; [2]Department of Pharmacy, The Fifth Affiliated Hospital of Sun Yat-sen University, Zhuhai, Guangdong Province 519000, China; [3]Rheumatology and Immunology Department, The Fifth Affiliated Hospital of Sun Yat-sen University, Zhuhai, Guangdong Province 519000, China

Systemic lupus erythematosus (SLE) is a complex autoimmune disease characterized by abnormal activation of B cells. Excessive B cell activating factor (BAFF) of the tumor necrosis factor family plays an essential role in B cell hyperactivation. Blocking the binding of excessive BAFF to its receptors is becoming a promising approach for SLE treatment. Here, we constructed genetically engineered exosomes derived from HEK293 cells that were functionalized with anti-CD19 (a B cell-specific surface marker) and loaded with anti-BAFF mRNA (CD19@Anti-BAFF^{mRNA} Exo). The anti-CD19 modification mediated receptor-dependent targeted binding and internalization, ensuring precise delivery of anti-BAFF mRNA to B cells. This enabled B cells to produce anti-BAFF antibodies *in vivo*, which captured excess BAFF, thereby inhibiting abnormal B cell activation and proliferation. Notably, mice with lupus treated with CD19@Anti-BAFF^{mRNA} Exo exhibited alleviated kidney damage and reduced disease progression. Our B cell-targeted engineered exosomes loaded with anti-BAFF mRNA provide a novel approach to enhance antibody production and improve local antibody concentration, offering a potential therapeutic strategy for SLE.

INTRODUCTION

Systemic lupus erythematosus (SLE) is a chronic autoimmune disease characterized by dysregulated B cell activity, autoantibody production, and multiorgan inflammation.[1] Traditional small-molecule chemical drugs have a wide range of therapeutic mechanisms of action; however, long-term use of these agents can easily lead to serious adverse reactions.[2] Therefore, novel biological agents have emerged as an important a new direction in SLE treatment.

B cells play a central role in SLE pathogenesis by producing pathogenic autoantibodies (e.g., anti-double-stranded DNA [dsDNA]) and secreting proinflammatory cytokines such as interleukin-6 (IL-6) and interferon-γ (IFN-γ).[3] Abnormal B cell immune tolerance leads to impaired elimination of autoreactive B cells, resulting in sustained autoantibody production.[4,5] Among the identified therapeutic targets, B cell activating factor (BAFF) has been recognized as an effective target.[6] BAFF is a typical B cell co-stimulatory molecule that promotes apoptosis resistance, class-switch recombination, and differentiation of autoreactive B cells into antibody-producing cells.[7] BAFF serves as a pivotal determinant of B cell survival, proliferation, and differentiation of B cells[8] Upon binding to its distinct receptor subtypes, BAFF modulates cytokine secretion through multiple complex signaling cascades.[9] Moreover, BAFF promotes an imbalance in T helper 1/T helper 2 (Th1/Th2) cell polarization by increasing Th1 cytokines,[10] thereby exacerbating SLE.[11] In patients with SLE, the elevated levels of serum-soluble BAFF are associated with disease activity and are strongly linked to disease pathogenesis.[12]

BAFF-targeting drugs (e.g., belimumab) have been approved for treatment of both pediatric and adult SLE,[13,14] but the clinical response rate to belimumab is only approximately 53.8%,[15] which may be partly attributable to the susceptibility of monoclonal antibodies to degradation *in vivo* and their limited ability to reach target cells effectively. Prolonging antibody activity or enabling sustained antibody secretion *in vivo* may therefore represent key strategies to improve therapeutic efficacy.

The strategy of employing mRNA delivery for antibody generation has emerged as a promising approach,[16] initially applied in the treatment of viral infection.[17] With the progressive maturation of this technology, it has been increasingly applied to antibody production and chimeric antigen receptor T (CAR-T) cell production *in vivo*.[18,19] To protect mRNA from degradation by various nucleases in blood

Received 7 May 2025; accepted 27 January 2026;
https://doi.org/10.1016/j.ymthe.2026.01.037.

[4]These authors contributed equally

Correspondence: Yongjian Wu, Center for Infection and Immunity and Guangdong Provincial Engineering Research Center of Molecular Imaging, The Fifth Affiliated Hospital of Sun Yat-sen University, Zhuhai, Guangdong Province 519000, China.
E-mail: wuyj228@mail.sysu.edu.cn
Correspondence: Xi Huang, Center for Infection and Immunity and Guangdong Provincial Engineering Research Center of Molecular Imaging, The Fifth Affiliated Hospital of Sun Yat-sen University, Zhuhai, Guangdong Province 519000, China.
E-mail: huangxi6@mail.sysu.edu.cn

© 2026 The Authors.
Published by Elsevier Inc. on behalf of The American Society of Gene and Cell Therapy.
This is an open access article under the CC BY license (http://creativecommons.org/licenses/by/4.0/).

and cells,[20] exosomes have emerged as an efficient delivery system due to their natural biocompatibility, low immunogenicity, and capacity for cell-specific targeting through surface modifications.[21,22] In addition, exosomes exhibit strong potential for extensive engineering, enabling functions such as the active loading of mRNA and the targeting of B cells.[23,24] As a B cell-specific surface marker, CD19 represents an ideal targeting ligand for mediating precise delivery of exosomes to B cells, and it has been widely used in B cell-targeted therapeutic strategies.[25] Therefore, the use of using engineered exosomes to deliver mRNA for antibody generation may enhance antibody production efficiency by enabling sustained antibody secretion at pathogenic sites.

In this study, we constructed B cell-targeted exosomes loaded with anti-BAFF mRNA (CD19@Anti-BAFF^{mRNA} Exo), which enabled B cells to produce anti-BAFF antibodies *in vivo*. By binding to the excessive BAFF in SLE, CD19@Anti-BAFF^{mRNA} Exo inhibited abnormal B cell activation and proliferation. In addition, we demonstrated that lupus-prone mice treated with CD19@Anti-BAFF^{mRNA} Exo exhibited alleviated lupus-related manifestations. Collectively, these findings indicate that the engineered exosome system enhances anti-BAFF antibody production and improves local antibody availability, thereby providing a potential therapeutic strategy for SLE.

RESULTS

Preparation and characterization of CD19@Anti-BAFF^{mRNA} Exo

To validate our hypothesis, we engineered exosomes expressing a single-chain variable fragment (scFv) derived from antibodies recognizing CD19 as well as mRNA encoding an scFv targeting BAFF. Specifically, three plasmids were designed to generate the engineered exosomes (Figures 1A and 1B). The plasmid expressing anti-CD19 scFv (pLV-1D3) contained a CD8α signal peptide, the anti-CD19 scFv sequence, a Myc tag, and a transmembrane domain. To facilitate efficient mRNA loading into exosomes, pLV-CD63-L7Ae, which enables RNA binding, and pLV-anti-BAFF-C/Dbox, which encodes the corresponding mRNA, were constructed. The pLV-CD63-L7Ae plasmid encoded the transmembrane protein CD63 fused to the RNA-binding peptide L7Ae, whereas the pLV-anti-BAFF-C/Dbox plasmid contained a CD8α signal peptide, the anti-BAFF scFv, a FLAG tag, and the C/Dbox structure capable of binding L7Ae. Lentiviral vectors were generated by cloning these constructs and transfecting them into HEK293T cells, followed by lentiviral infection and stable cell line selection (Figure S1). HEK293T cells were used as exosome-producing cells for all subsequent experiments owing to their ease of handling, high transfection efficiency, and robust exosome production capacity.

By transfecting different plasmid combinations, four stable HEK293T cell lines were generated, from which four corresponding exosome populations were isolated from culture supernatants via differential ultracentrifugation (UC): free Exo (G1) without modification, CD19 Exo (G2) with anti-CD19 modification targeting B cells, anti-BAFF^{mRNA} Exo (G3) with anti-BAFF mRNA modification producing anti-BAFF, and CD19@Anti-BAFF^{mRNA} Exo (G4) with both anti-CD19 and anti-BAFF mRNA modification possessing both abilities. To detect the characterization of these prepared exosomes, transmission electron microscopy (TEM) confirmed that they were homogeneous with a round or oval morphology (Figure 1C). Nanoparticle tracking analysis (NTA) showed that the modal particle size of CD19@Anti-BAFF^{mRNA} Exo was 149.4 ± 3.3 nm (Figure 1D), and comparable size distributions were observed among the different exosome groups (Figure 1E). The zeta potentials varied among groups, likely reflecting differences in surface modifications (Figure 1F).

Protein characterization of CD19@Anti-BAFF^{mRNA} Exo, including the assessment of exosome biomarkers, was performed using western blotting. According to the International Society for Extracellular Vesicles MISEV2023 (Minimal Information for Studies of Extracellular Vesicles) guidelines,[26] exosome characterization should include at least three positive EV markers, including one transmembrane or lipid-bound protein (e.g., CD63) and one cytosolic protein (e.g., TSG-101), as well as the absence of negative markers such as calnexin. Consistent with these criteria, CD81, TSG101, and CD63 were detected, whereas the endoplasmic reticulum marker calnexin was absent in CD19@Anti-BAFF^{mRNA} Exo (Figure 1G). This observation underscores the quality of the exosomes and the absence of endoplasmic reticulum contamination. Furthermore, detection of anti-CD19 in the exosomes indicated successful surface modification, while the presence of anti-BAFF demonstrated effective loading of the therapeutic cargo (Figure 1H). The increased abundance of anti-BAFF mRNA within exosomes further confirmed that the mRNA was successfully encapsulated (Figure 1I). Collectively, these results demonstrate that the engineered exosomes possessed the expected physicochemical characteristics, expressed surface anti-CD19, and encapsulated mRNA within them simultaneously.

CD19@Anti-BAFF^{mRNA} Exo specifically target B cells and induce production of anti-BAFF antibodies

As previously mentioned, although we have demonstrated the potential of CD19@Anti-BAFF^{mRNA} Exo to target B cells (mediated by anti-CD19) and carry anti-BAFF mRNA, their actual functional effects require further investigation and verification. To detect the targeting capability *in vitro*, different groups of exosomes stained with DiO were cultured with B cells isolated from mouse spleens. Flow cytometry showed that anti-CD19-modified exosomes exhibited an increased uptake rate by B cells (Figure 2A). Meanwhile, confocal microscopy revealed that exosomes modified with anti-CD19 (G2 and G4) were phagocytosed by B cells more efficiently than those without targeting modification (G1 and G3) (Figure 2B). To further explore the targeting capability of CD19@Anti-BAFF^{mRNA} Exo *in vivo*, biodistribution was monitored using an *in vivo* imaging system (IVIS) at 24 h post-intravenous injection. While DiR-labeled exosomes mainly accumulated in the liver, exosomes modified with anti-CD19 (G2 and G4), unlike the unmodified groups (G1 and G3), showed additional enrichment in the spleen, demonstrating B cell tropism (Figure 2C).

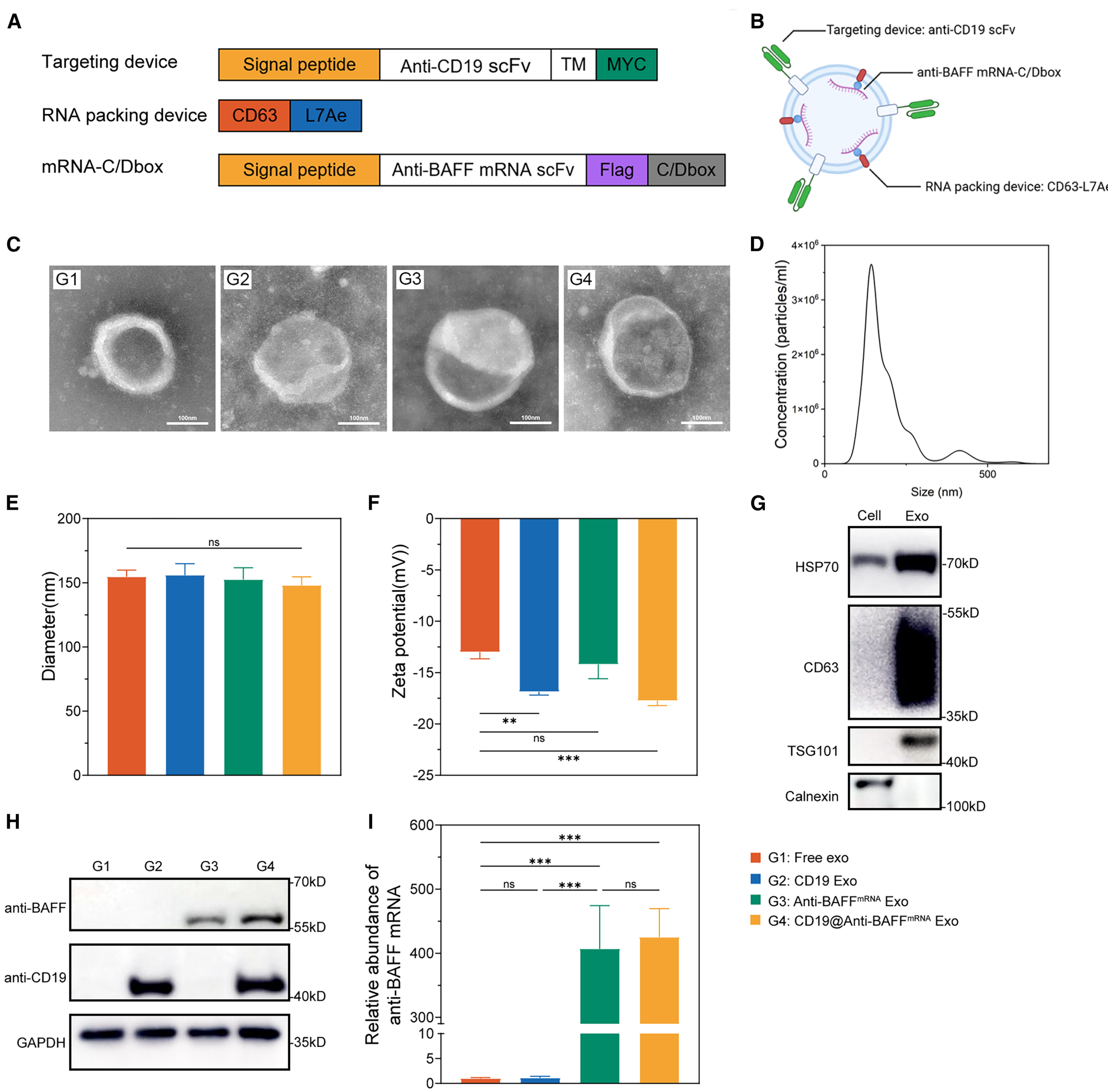

Figure 1. Preparation and characterization of CD19@Anti-BAFF^{mRNA} Exo

(A) Molecular design of anti-CD19, RNA packaging device, and anti-BAFF^{mRNA}. (B) Schematic diagram of CD19@Anti-BAFF^{mRNA} Exo. (C) Representative TEM image of exosomes from each group. Scale bar: 100 nm. (D) NTA was used to determine the size distribution of CD19@Anti-BAFF^{mRNA} Exo. (E and F) Hydrodynamic diameter (E) and zeta potential (F) of exosomes in each group were measured by dynamic light scattering. (G) The expression of exosome markers in whole-cell lysates of CD19@Anti-BAFF^{mRNA} cells or exosomes was analyzed by western blotting. (H) The expression of anti-CD19 and anti-BAFF in each group was analyzed by western blotting. (I) The relative abundance of anti-BAFF mRNA was analyzed by quantitative RT-qPCR. Data are presented as the mean ± SEM of three biological replicates. *$p < 0.05$; **$p < 0.01$; ***$p < 0.005$; ns, not significant.

Meanwhile, the efficiency of CD19@Anti-BAFF^{mRNA} Exo in inducing anti-BAFF antibody production by B cells was evaluated both *in vitro* and *in vivo*. Initially, different groups of exosomes were incubated with B cells isolated from mouse spleens. Western blot analysis revealed the presence of anti-BAFF-FLAG in the G3 and G4 groups containing anti-BAFF^{mRNA} modification (Figure 2D). In addition, exosomes at various concentrations were co-incubated with B cells, and the samples were subsequently

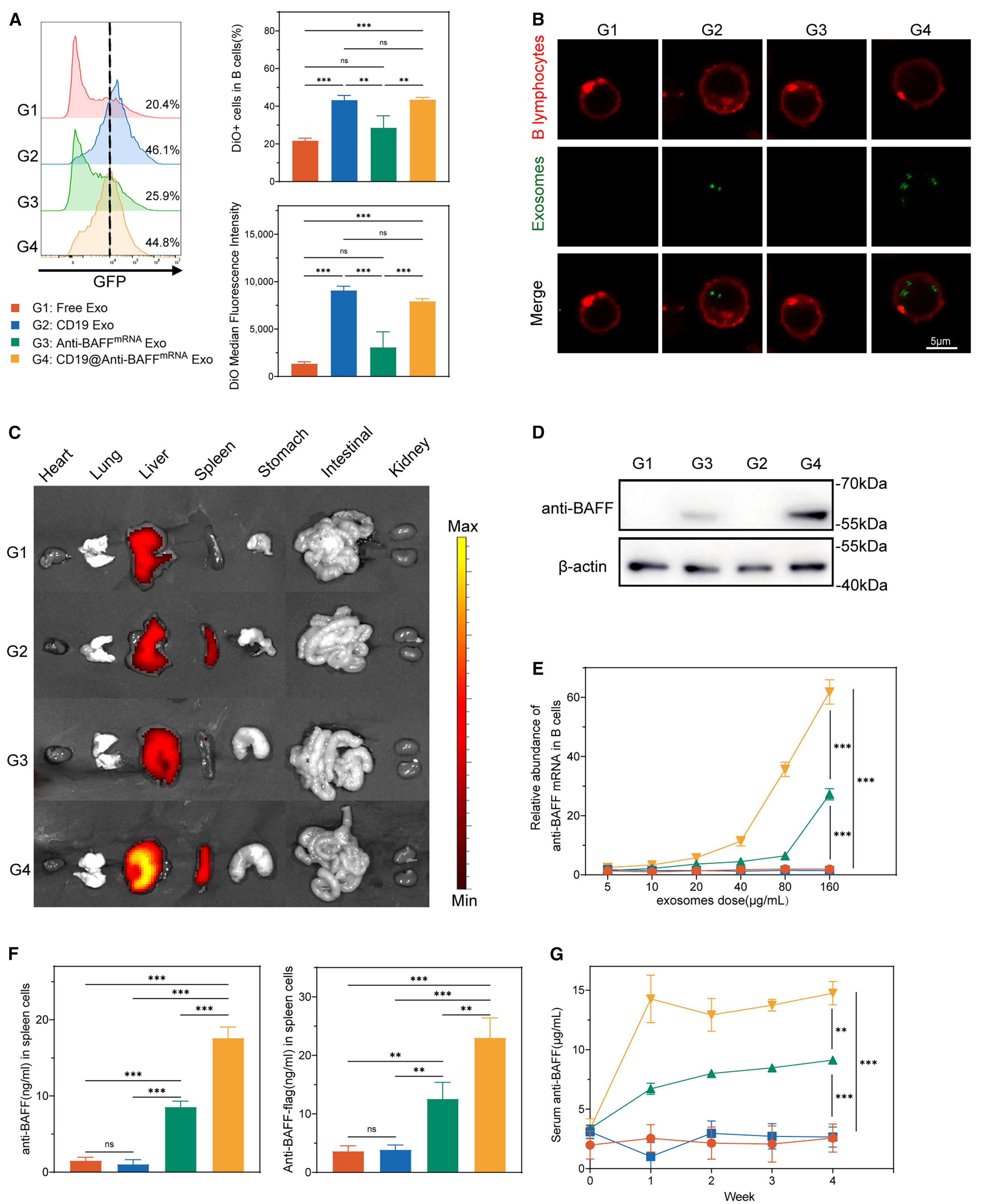

(legend on next page)

analyzed by RT-qPCR. The results clearly demonstrated that antibody production increased in a dose-dependent manner with increasing exosome concentration (Figure 2E). Furthermore, ELISA assays targeting FLAG and anti-BAFF showed that anti-BAFF antibody production occurred in the supernatants of the G3 and G4 groups treated with exosomes modified anti-BAFF^{mRNA}. Moreover, due to the targeting effect, the antibodies induced by CD19@Anti-BAFF^{mRNA} Exo were approximately 2-fold higher than those induced by anti-BAFF^{mRNA} Exo alone (Figure 2F).

To investigate antibody production *in vivo*, exosomes from each group were intravenously administered twice per week, and serum samples were collected weekly for anti-BAFF-FLAG ELISA analysis. As shown in Figure 2G, serum anti-BAFF antibody levels peaked during the first week and were maintained with subsequent administrations. Consistent with the *in vitro* findings, the efficiency of anti-BAFF antibody production following CD19@Anti-BAFF^{mRNA} Exo treatment was higher than that achieved with anti-BAFF^{mRNA} Exo alone, highlighting the advantage of the targeted delivery strategy.

These findings demonstrate that CD19@Anti-BAFF^{mRNA} Exo specifically target B cells and effectively induce anti-BAFF antibody production, as anticipated.

CD19@Anti-BAFF^{mRNA} Exo inhibited the activation and proliferation of B cells

Moreover, we detected changes in cytokine levels in mouse splenic cells co-incubated with different groups of exosomes under BAFF stimulation to further explore the effects of CD19@Anti-BAFF^{mRNA} Exo on B cell activation. The results showed that the levels of pro-inflammatory cytokines IL-1β, IL-6, and tumor necrosis factor α (TNF-α) in spleen cells were markedly reduced compared with those in other groups (Figure 3A). Meanwhile, the expression levels of cytokines associated with the Th1/Th2 axis, including IL-4 and the p40 subunit of IL-12 secreted by Th1 cells, were also decreased in spleen cells treated with CD19@Anti-BAFF^{mRNA} Exo (Figure 3B). In contrast, the expression of immunosuppressive cytokines, such as IL-10 and transforming growth factor β (TGF-β), was significantly upregulated (Figure 3C). These results indicated that CD19@Anti-BAFF^{mRNA} Exo inhibited cytokines that promote B cell activation while they simultaneously enhanced the release of anti-inflammatory cytokines, thereby suppressing B cell activation.

Moreover, flow cytometry analysis showed that in BAFF-stimulated spleen cells treated with CD19@Anti-BAFF^{mRNA} Exo, the proportions of major histocompatibility complex (MHC) class II, CD69, and CD80 on CD19^{+} B cells were decreased compared with those in other groups, further indicating inhibition of B cell activation (Figure 3D). In addition, under BAFF stimulation, carboxyfluorescein succinimidyl ester (CFSE)-labeled mouse splenic cells were co-incubated with different groups of exosomes for 5 days. Flow cytometric analysis demonstrated that B cell proliferation was inhibited in the CD19@Anti-BAFF^{mRNA} Exo-treated group (Figure 3E). Furthermore, western blot analysis confirmed that the activation of the nuclear factor κB and mammalian target of rapamycin signaling pathways induced by BAFF was suppressed by CD19@Anti-BAFF^{mRNA} Exo (Figures 3F and 3G). In conclusion, CD19@Anti-BAFF^{mRNA} Exo generated anti-BAFF antibodies that neutralized excess BAFF and subsequently inhibited B cell activation and proliferation.

CD19@Anti-BAFF^{mRNA} Exo alleviated the symptoms of lupus in mice induced by ALD-DNA

As described above, we verified the targeting ability of exosomes to B cells and confirmed the generation of anti-BAFF antibodies. To further investigate the therapeutic effects of CD19@Anti-BAFF^{mRNA} Exo under pathologic conditions, we established an activated lymphocyte derived (ALD)-DNA-induced lupus mouse model and randomly divided the mice into four treatment groups receiving different exosome formulations (Figure 4A). We initially determined that the half-lives of CD19@Anti-BAFF^{mRNA} Exo and the encapsulated mRNA in the spleens of C57 mice were approximately 12–24 h and 48–72 h, respectively (Figure S2). Accordingly, we administered exosomes twice per week. Reduced serum levels of anti-dsDNA antibodies and antinuclear antibodies (ANAs) were found in mice treated with CD19@Anti-BAFF^{mRNA} Exo (Figures 4B and 4C), with significantly decreased urinary protein (Upro) levels (Figure 4D). The size and weight of the spleens and lymph nodes in the CD19@Anti-BAFF^{mRNA} Exo treatment group were also significantly smaller than those of the other groups (Figures 4E and 4F).

We also detected the histologic damage and the deposition of complement in mice with lupus, reflecting disease remission. The results of hematoxylin and eosin (H&E) and Masson staining revealed that CD19@Anti-BAFF^{mRNA} Exo treatment alleviated glomerular mesangial expansion (Figure 4G). Correspondingly, glomerulonephritis

Figure 2. CD19@Anti-BAFF^{mRNA} Exo specifically target B cells and induce production of anti-BAFF antibodies

(A) Flow cytometric analyses were conducted to assess the uptake of DiO-stained exosomes by B lymphocytes, with GFP fluorescence detected. (B) Representative confocal laser scanning microscopy images show exosomes bound to the surface of B lymphocytes (stained with DiI, red). Exosomes from each group were labeled with DiO (green). Exosomes modified with anti-CD19 (G2 and G4) exhibited a higher binding affinity to B lymphocytes compared with exosomes without anti-CD19 modification (G1 and G3). Scale bar: 10 μm. (C) Representative *in vivo* imaging system images show the biodistribution of DiR-labeled exosomes *in vivo* after intravenous injection. (D) The expression of anti-BAFF in the whole-cell lysates of B lymphocytes after co-incubation with exosomes from each group was analyzed by western blotting. (E) The relative abundance of anti-BAFF mRNA at different doses in each group was analyzed by RT-qPCR. (F) The expression of anti-BAFF antibodies in the supernatants of spleen cells after co-incubation with exosomes from each group was quantified by ELISA. (G) The expression levels of anti-BAFF antibodies in the serum of mice after intravenous injection of exosomes from each group were quantified by ELISA. Data are presented as the mean ± SEM of three biological replicates. *$p < 0.05$; **$p < 0.01$; ***$p < 0.005$; ns, not significant.

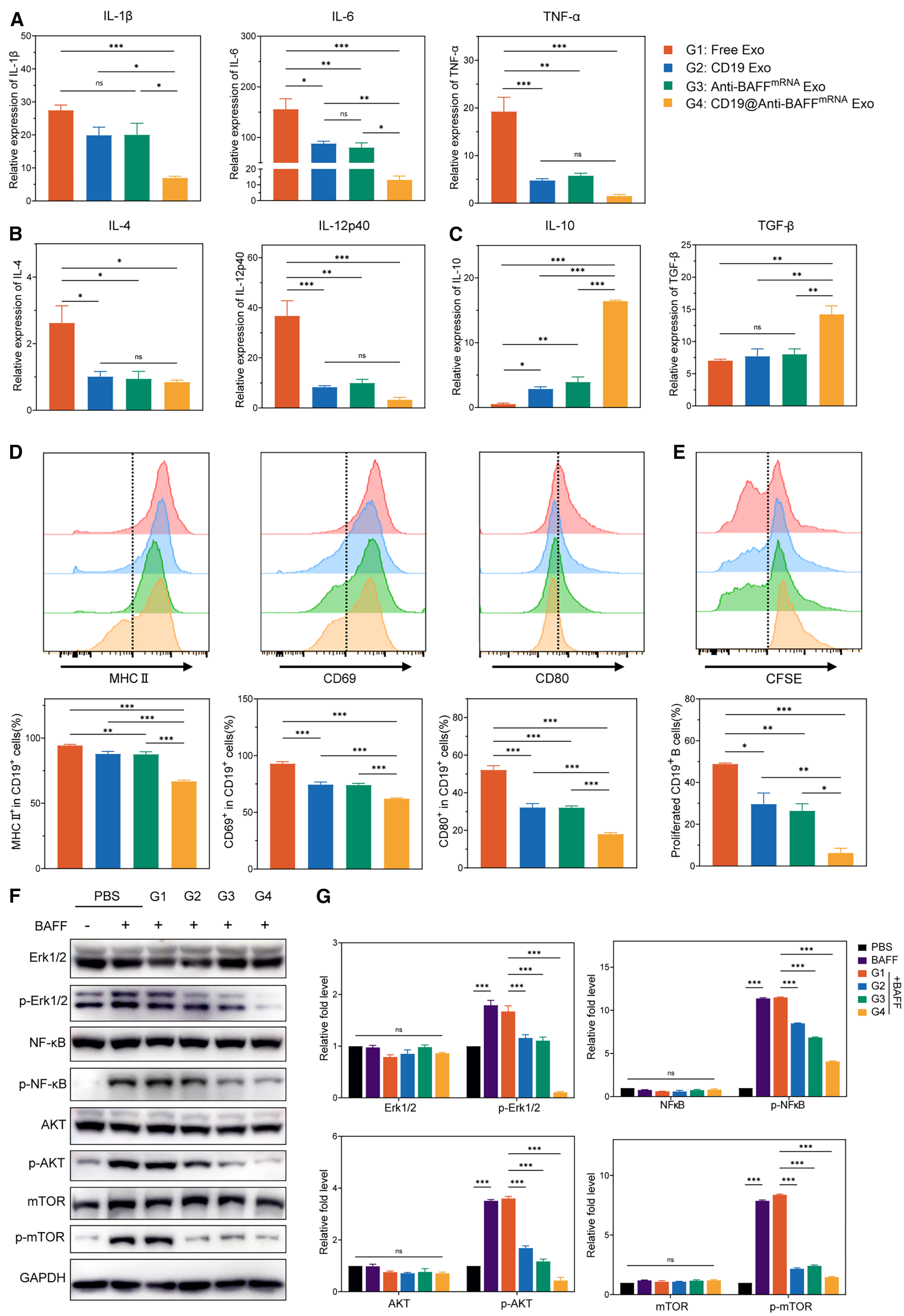

(legend on next page)

(GN) scores showed a significant reduction in renal injury in mice treated with CD19@Anti-BAFF^{mRNA} Exo (Figure 4H). Immunofluorescence staining further confirmed that CD19@Anti-BAFF^{mRNA} Exo treatment decreased the immunological deposits of immunoglobulin G (IgG) and C3 in the kidneys (Figures 4I and 4J). Collectively, these findings demonstrate that CD19@Anti-BAFF^{mRNA} Exo effectively alleviated lupus manifestations in ALD-DNA-treated mice.

Immune cell populations in the spleen were analyzed further by flow cytometry. Compared with the free Exo group (G1), the frequencies of B cells were reduced in mice treated with the other exosome formulations (G2–G4) (Figure 5A). Moreover, treatment with engineered exosomes resulted in decreased frequencies of memory B cells (Figure 5B), plasma cells (Figure 5C), and plasmablasts (Figure 5D), along with an increased proportion of naive B cells (Figure 5B). Among the groups, CD19@Anti-BAFF^{mRNA} Exo exhibited the most pronounced effects, reflecting its combined targeting and antibody-inducing capabilities. In addition, the frequency of follicular helper T (Tfh) cells was reduced in the spleens of mice treated with CD19@Anti-BAFF^{mRNA} Exo, indicating attenuation of SLE-associated immune activation (Figure 5E).

Furthermore, analysis of splenic macrophages, dendritic cells, monocytes, and neutrophils revealed no statistically significant differences among the experimental groups (Figure S3). These data indicated a decrease in autoreactive B cells and Tfh cells promoting autoimmune activation, along with the generation of newly formed naive B cells, collectively suggesting an improvement in the lupus condition.

CD19@Anti-BAFF^{mRNA} Exo alleviated the symptoms of MRL/Lpr mice

Although the therapeutic effect of CD19@Anti-BAFF^{mRNA} Exo was confirmed in lupus mice induced by ALD-DNA, we further evaluated its efficacy in lupus-prone MRL/Lpr mice. MRL/Mpj mice were used as the non-diseased negative control group, and tabalumab, an anti-BAFF monoclonal antibody,was used as a positive control to compare the therapeutic efficacy of engineered exosomes with direct antibody therapy. Typically, MRL/Lpr mice begin to develop lupus symptoms at approximately 12 weeks of age, and following disease onset, their lifespan rarely exceeds 18 weeks. Considering that most patients initiate treatment after disease onset, we selected the post-onset stage for therapeutic intervention (Figure 6A). After treatment, compared with MRL/Lpr mice receiving unmodified exosomes (G1), those treated with engineered exosomes (G4) exhibited a significantly prolonged survival time, which was also superior to that observed in MRL/Lpr mice treated with tabalumab (G5) (Figure 6B).

Compared with MRL/Mpj mice, MRL/Lpr mice treated with unmodified exosomes displayed pronounced lupus manifestations, including elevated autoantibody levels, increased Upro excretion, lymphoid organ enlargement, and severe renal injury. In contrast, MRL/Lpr mice treated with CD19@Anti-BAFF^{mRNA} Exo showed a significant reduction in anti-dsDNA, ANA, and Upro levels, approaching near-normal levels (Figures 6C and 6D). Levels of proinflammatory cytokines, including IL-6, TNF-α, and IFN-γ, which are elevated during lupus progression, were also significantly reduced following treatment to levels comparable to those in mice without disease (Figure 6E). Moreover, spleen and lymph node enlargement was substantially alleviated, as evidenced by decreases in organ size and weight (Figures 6F and 6G). Furthermore, CD19@Anti-BAFF^{mRNA} Exo treatment also led to a reduction in glomerular mesangial expansion, crescent formation, endothelial cell hyperplasia, and inflammatory cell infiltration in the kidney (Figure 6H). In addition, immunofluorescence analysis showed decreased deposition of IgG and C3 in the kidneys of MRL/Lpr mice (Figure 6I). Although treatment with tabalumab also ameliorated lupus symptoms and renal injury, its therapeutic effect was less pronounced than that observed with CD19@Anti-BAFF^{mRNA} Exo.

The immunological profiles of splenic immune cells were analyzed further by flow cytometry. Compared with the free Exo group (G1), a reduced frequency of B cells was observed in mice treated with the other exosomes and tabalumab in G2–G5 (Figure 7A). Furthermore, treatment with engineered exosomes led to a decrease in the frequencies of memory B cells (Figure 7B), plasma cells (Figure 7C), and plasmablasts (Figure 7D), accompanied by an increase in naive B cells (Figure 7B). Among T cell subsets, analysis revealed reduced frequencies of Tfh cells and Th17 cells, along with an increased proportion of regulatory T cells (Tregs) In the spleens of MRL/Lpr mice treated with CD19@Anti-BAFF^{mRNA} Exo, a reduced frequency of Tfh cells and Th17 cells and an increased frequency of Tregs were also observed (Figures 7E–7G). CD19@Anti-BAFF^{mRNA}

Figure 3. CD19@Anti-BAFF^{mRNA} Exo inhibited the activation and proliferation of B cells

BAFF-stimulated mouse spleen cells were treated with free Exo, CD19 Exo, anti-BAFF^{mRNA} Exo, and CD19@Anti-BAFF^{mRNA} Exo *in vitro*. (A) The mRNA expression levels of pro-inflammatory factors (including IL-1β, IL-6, and TNF-α) in B cells after treatment with different groups. (B) The mRNA expression levels of cytokines related to the Th1/Th2 axis, IL-4 and IL-12p40. (C) The mRNA expression levels of anti-inflammatory factors (including IL-10 and TGF-β). (D) Representative flow cytometry images and statistical analysis of the proportions of MHC class II$^+$, CD69$^+$, and CD80$^+$ cells in CD19$^+$ B cells treated with different groups. (E) Representative flow cytometry images (top) and statistical analysis (bottom) of the proportions of proliferating CD19$^+$ B cells treated with different groups. Data are presented as the mean ± SEM of three biological replicates. (F) Representative western blot images showing the expression levels of total proteins (ERK1/2, nuclear factor κB [NF-κB], AKT, mammalian target of rapamycin [mTOR]) and their phosphorylated isoforms (p-ERK1/2, p-NF-κB, *p*-AKT, and p-mTOR) in BAFF-stimulated splenocytes following co-incubation with various exosome groups. Splenocytes were pretreated with 100 ng/mL BAFF and then co-cultured with exosomes for 24 h. GAPDH was used as the loading control. (G) Gray value quantification was performed using ImageJ software, and data were normalized to the internal reference protein. Data are presented as the mean ± SEM of three biological replicates. *$p < 0.05$; **$p < 0.01$; ***$p < 0.005$; ns, not significant.

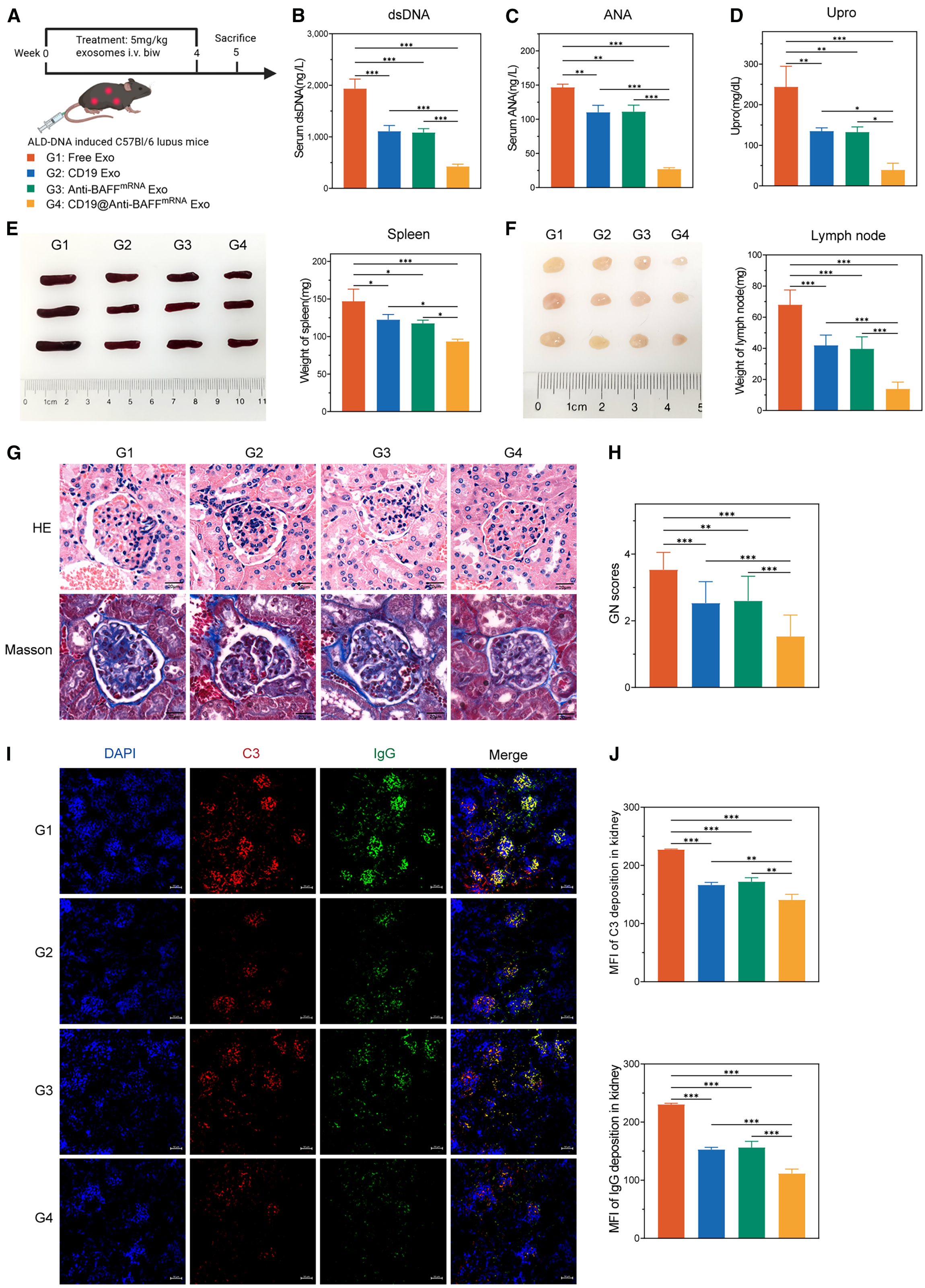

(legend on next page)

Exo demonstrated the most significant effect among these exosomes with different modifications and exhibited efficacy superior to that of tabalumab. Hence, in MRL/Lpr mice, CD19@Anti-BAFF^{mRNA} Exo also exerted a favorable therapeutic effect and alleviated the symptoms of lupus. Moreover, this therapeutic effect was superior to that of tabalumab.

To further assess the biocompatibility of the engineered exosomes, major organs (heart, liver, spleen, lung, and kidney) from wild-type mice treated with CD19@anti-BAFF^{mRNA} Exo were examined. No apparent histopathologic abnormalities were observed, and no significant alterations in major biochemical parameters were detected (Figure S4). Collectively, these results demonstrate that CD19@Anti-BAFF^{mRNA} Exo possesses favorable biocompatibility and therapeutic efficacy, supporting its potential for further translational development.

DISCUSSION

Currently, the etiology of SLE remains unclear, and therapeutic strategies are therefore primarily focused on inhibiting aberrant immune activation to alleviate symptoms. Compared with first-line glucocorticoids, biologics that block pathogenic B cell antibody production have been extensively studied and applied due to their higher targeting specificity and fewer systemic side effects. Clinical studies have demonstrated that currently available biologics can improve lupus symptoms and prognosis in patients with moderate to severe disease, showing promising therapeutic potential.[27] Belimumab, the first approved biologic with the longest clinical application history, offers relatively reliable safety guarantees.[28] Belimumab can reduce patients' dependence on glucocorticoids.[29] However, its therapeutic efficacy remains limited due to incomplete clearance of pathogenic B cells, restricted applicability to certain patient populations, disease severity, and other factors.[30] This limitation may be associated with the insufficient responsiveness of certain pathogenic B cell subsets to BAFF blockade. In addition, evidence suggests that combining BAFF antagonism therapy with other therapeutic strategies can further improve patient prognosis.[31] From the perspective of optimizing therapeutic efficacy, compared with systemic anti-BAFF therapy, the exosome-based delivery strategy employed in this study not only enhances targeting specificity, enabling more precise delivery of anti-BAFF agents to B cells, but also exerts a unique cascade amplification effect. Moreover, SLE is a chronic autoimmune disease requiring long-term management, and exosome-based approaches exhibit lower immunogenicity than repeated monoclonal antibody administration during acute exacerbations. Compared with monotherapeutic antibody therapy, exosomes exhibit significantly lower immunogenicity, thereby reducing the risk of adverse immune rejection.[32]

To address the incomplete B cells observed with BAFF antibodies, CD19 and CD20 have been widely explored as alternative or complementary therapeutic targets in SLE. Studies have demonstrated that sequential administration of anti-CD20 agents combined with belimumab exerts synergistic therapeutic effects in refractory SLE.[33] However, despite its clinical use, rituximab monotherapy has shown limited efficacy in SLE, and the underlying mechanisms remain incompletely understood. Compared with CD20, CD19 is more broadly expressed across the entire B cell lineage, and accumulating evidence indicates that CD19^{+} plasma cells play a critical role in the production of pathogenic antibodies.[34] CD19 antagonism effectively reduces serum autoantibody levels in SLE mouse models,[35] and CD19-directed antibody-drug conjugates have also demonstrated promising therapeutic potential in SLE treatment.[36] In the present study, we demonstrated that engineered exosomes expressing CD19-targeting scFv can alleviate disease manifestations in SLE models and synergize with BAFF inhibition. Serologic analyses further supported this finding, indicating that CD19-mediated targeting enhances the clearance of pathogenic B cells and improves the efficacy of BAFF-directed therapy. These results suggest that this combinatorial strategy represents a promising approach for SLE treatment.

mRNA-based therapy has emerged as a highly promising platform for *in vivo* protein production approach.[37] Its advantages include rapid production, scalable manufacturing, and the capacity for repeated administration, making it particularly suitable for the treatment of chronic diseases.[38] However, the intrinsic instability of mRNA limits its therapeutic application. Delivery systems are therefore required to protect mRNA from degradation and facilitate efficient cellular uptake. Compared with conventional lipid nanoparticle (LNP)-based systems, exosome-mediated mRNA delivery offers superior biocompatibility, reduced immunogenicity, and enhanced biological compatibility, enabling repeated administration with improved safety profiles. Nevertheless, the low mRNA loading efficiency of conventional exosomes prevents achieving effective therapeutic concentrations. Therefore, improving the mRNA loading capacity of exosomes is crucial. Here, we modified anti-BAFF scFv mRNA with the L7Ae-binding C/D box to enhance exosome loading efficiency.[23] We verified the high efficiency of this system in loading mRNA into exosomes and confirmed its favorable delivery and therapeutic effects in preclinical models. These findings further validate the prospects of exosome-mediated mRNA CAR-T cell therapy.

Figure 4. CD19@Anti-BAFF^{mRNA} Exo alleviated the symptoms of lupus mice treated by ALD-DNA

(A) Schematic diagram depicting the experimental design for evaluating the efficacy of exosomes from each group in treating lupus mice induced by ALD-DNA. Mice were intravenously injected twice per week for 1 month. (B–D) The levels of dsDNA (B) and ANA (C) in serum and urinary protein (D) were quantified by ELISA. (E and F) Representative images of the spleens (E) or lymph nodes (F) from each group and statistical analysis of their weights. (G) Representative images of H&E-stained and Masson-stained kidney sections from each group. Scale bar: 20 μm. (H) Kidney pathology was assessed by GN scores. (I) Representative images of C3 (red) and IgG (green) deposition in the kidneys. Scale bar: 50 μm. (J) MFI of C3 and IgG deposition in the kidneys. Data are presented as the mean ± SEM of three biological replicates. *$p < 0.05$; **$p < 0.01$; ***$p < 0.005$; ns, not significant.

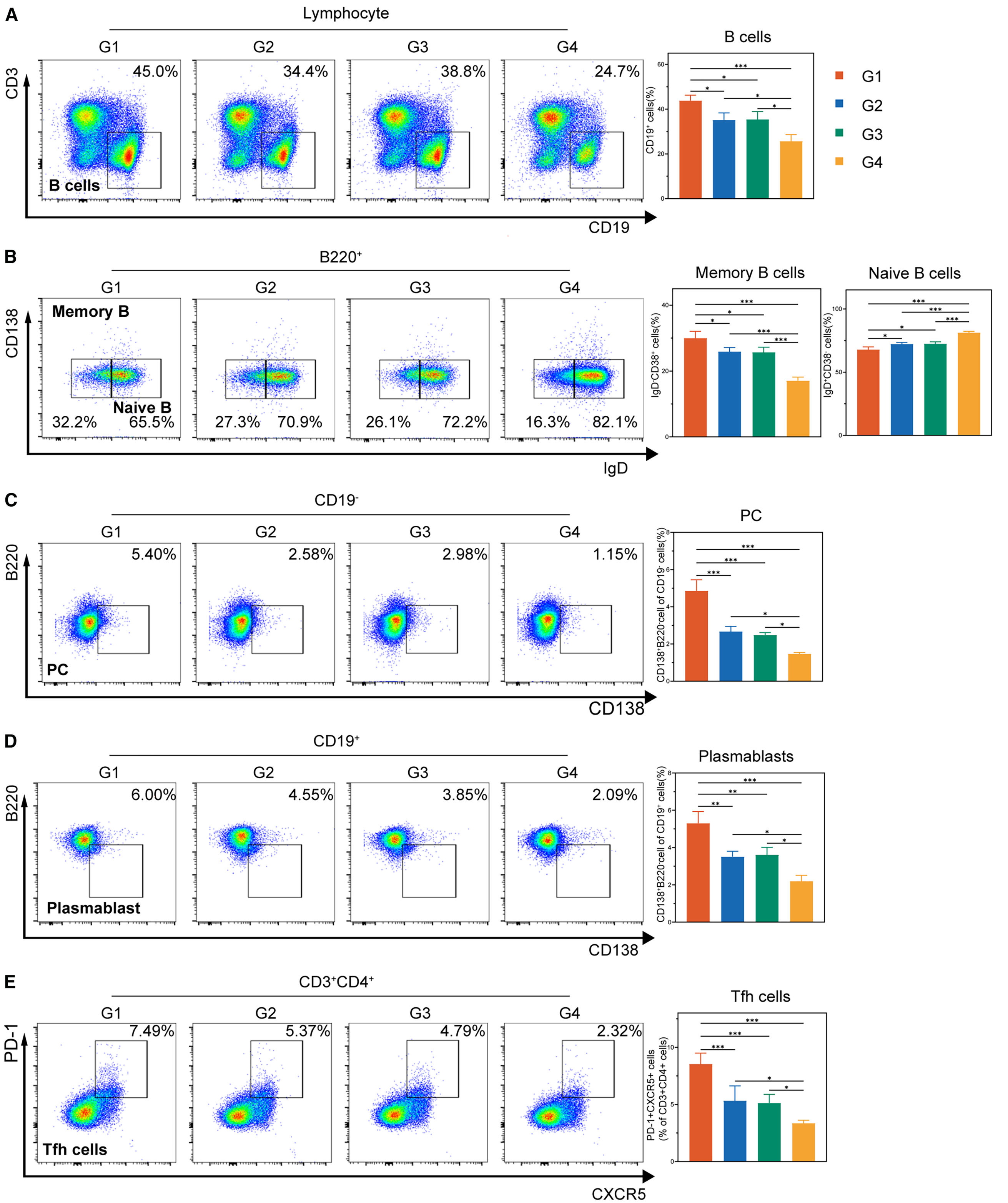

(legend on next page)

CAR-T therapy has achieved considerable success in the treatment of autoimmune diseases. Anti-CD19 CAR-T cells have demonstrated potent clearance of pathogenic B cells in MRL/Lpr mice and effectively ameliorated renal damage.[39] Dual-target CAR-T strategies targeting both CD19 and B cell maturation antigen can effectively relieve acute-phase symptoms in SLE patients,[40] highlighting their potential. However, CAR-T therapies are associated with substantial risks, including cytokine release syndrome (CRS), immune effector cell-associated neurotoxicity syndrome, and excessive immune suppression. Additionally, the requirement for personalized customization inadvertently increases the treatment burden on patients.[41] In contrast, exosome-based therapies offer several advantages and have shown great potential in clinical studies across multiple fields, with core strengths that include natural targeting ability, tissue penetration, low immunogenicity, high safety, and synergistic therapeutic potential. Xie et al. developed novel mesenchymal stem cell-derived exosomes that effectively alleviate symptoms in patients with pulmonary fibrosis.[42] Hadizadeh et al. developed umbilical cord-derived exosomes that efficiently promote fistula closure in Crohn disease patients, demonstrating promising clinical translation prospects.[43] Nevertheless, the translational application of exosome therapy is limited by several common challenges, such as batch effects and the high cost of large-scale production.[44] However, via future engineering modifications to enhance targeting ability, customize exosome cargo, and innovate production processes, exosomes are expected to become a new generation of precision therapeutic tools, holding substantial promise for patients with refractory diseases worldwide.

Bispecific antibodies (BsAbs) play a pivotal role in the development of therapies for SLE. For instance, telitacicept, which simultaneously targets BAFF and a proliferation-inducing ligand (APRIL), has demonstrated satisfactory efficacy in inhibiting the survival of abnormal B cells that secrete autoantibodies.[45] Another CD20-CD3 T cell-engaging bispecific monoclonal antibody, mosunetuzumab, can eliminate malignant B cells by redirecting T cells, and its phase 2 clinical trial results have shown promising therapeutic potential for SLE.[46] However, BsAbs still exhibit inherent limitations compared with targeted mRNA delivery systems. In contrast to the construction of anti-BAFF/anti-CD19 BsAbs, mRNA-targeted delivery strategies effectively circumvent the purification challenges associated with antibody production by enabling *in situ* antibody expression in B cell-enriched regions. The mRNA-LNP technology developed by Xu et al. shortened the monoclonal antibody production cycle from 6–8 months to 7 weeks, thereby significantly overcoming the efficiency limitations of traditional antibody preparation.[47] Additionally, the design of BsAbs is highly complex, requiring sophisticated equipment and prolonged development timelines from molecular design to completion of pharmacokinetic studies.[48] Therefore, the development of exosome-based targeted mRNA delivery systems represents a more cost-effective and practical alternative.

Another unique advantage of this strategy lies in its ability to enhance serum antibody concentrations and prolong circulatory half-life. Specifically, the LNP system loaded with SARS-CoV-2 neutralizing antibody mRNA developed by Deng et al. doubled serum neutralizing antibody concentrations compared with direct antibody infusion.[48] Furthermore, a study by Pardi et al. demonstrated that 24 h after tail vein injection of 30 μg rabiesvirus antibody mRNA-loaded LNPs, antibody levels in mouse serum far exceeded the half-maximal inhibitory concentration for HIV and remained detectable for up to 9 days.[49] These findings indicate that antibodies produced *in vivo* via targeted mRNA delivery systems exhibit superior durability compared with directly administered antibodies. Moreover, the CD19-targeting property of exosomes enables localized enrichment of BAFF antibodies within B cell-rich regions, which may represent a key mechanism underlying their enhanced therapeutic efficacy relative to monotherapy with antibodies.

Despite these advantages, the clinical application of BsAbs still faces significant challenges. In the treatment of B cell lymphoma, the incidence of CRS induced by CD20/CD3 BsAbs can reach as high as 26%.[50] CRS results from excessive immune activation and massive cytokine release, typically occurring within 48 h of initial administration.[51] In severe cases, excessive immune activation may also lead to neurologic toxicity, requiring intensive medical intervention and airway protection.[52] In contrast, endogenous antibodies produced via targeted mRNA delivery systems exhibit lower immunogenicity and induce fewer adverse reactions and are therefore more compatible with long-term therapeutic use in patients with SLE.

Despite the encouraging outcomes, several limitations remain. First, although CD19@Anti-BAFF^{mRNA} Exo targets B cells to induce anti-BAFF antibody production, thereby suppressing B cell activity, there remains a theoretical risk of excessive inhibition of normal B cell function. However, the dynamic expression profile of mRNA allows responsiveness to the local microenvironment; for example, in inflammatory settings, enhanced cellular activity may increase antibody production, forming a negative feedback loop that prevents excessive suppression of the normal immune function.[53] Therefore, further studies are required to determine optimal dosing regimens that achieve sufficient BAFF neutralization while preserving normal immune homeostasis. Second, the present study primarily evaluated

Figure 5. The phenotype of immune cells in mice with lupus induced by ALD-DNA after CD19@Anti-BAFF^{mRNA} Exo treatment

ALD-DNA-induced mice were divided into four groups and treated with exosomes with different modifications (G1: free Exo, G2: CD19 Exo, G3: anti-BAFF^{mRNA} Exo, G4: CD19@Anti-BAFF^{mRNA} Exo). The expression of cell-specific surface markers was detected by flow cytometry. Representative flow cytometry images and statistical analysis of the proportion of B cells ($CD3^{-}CD19^{+}$) (A), naive B cells ($B220^{+}IgD^{+}CD138^{-}$), memory B cells ($B220^{+}IgD^{-}CD138^{-}$) (B), plasma cells ($CD19^{-}B220^{-}CD138^{+}$) (C), plasmablasts ($CD19^{+}B220^{-}CD138^{+}$) (D), and Tfh cells ($CD3^{+}CD4^{+}PD\text{-}1^{+}CXCR5^{+}$) (E) in spleens. Data are presented as the mean ± SEM of three biological replicates. *$p < 0.05$; **$p < 0.01$; ***$p < 0.005$; ns, not significant.

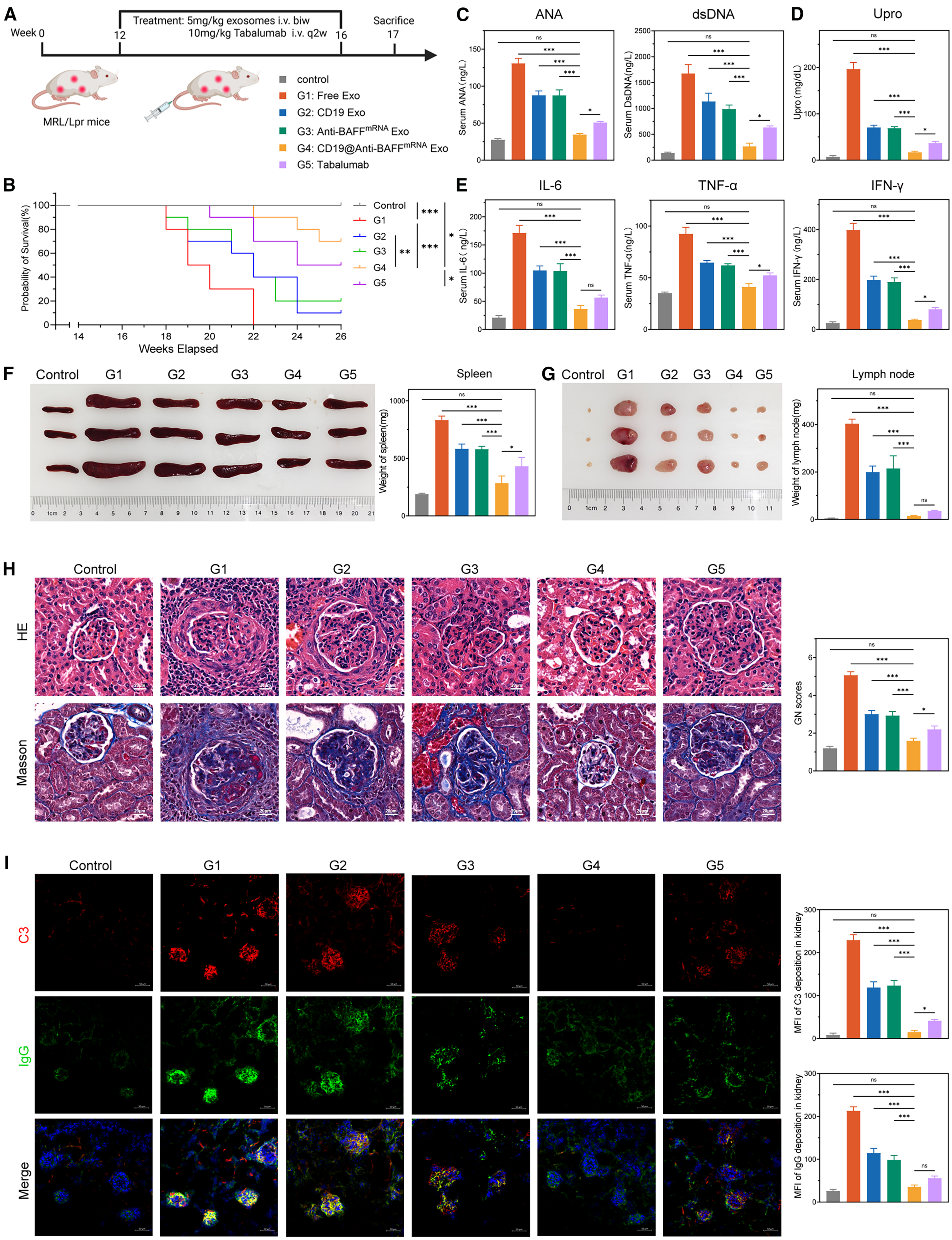

(legend on next page)

short-term therapeutic outcomes, and long-term efficacy and safety remain to be fully elucidated. Extended observation periods will be necessary to assess sustained therapeutic benefit and potential adverse effects. In addition, large-scale production and quality control of exosomes remain challenging, as variability in mRNA loading efficiency may influence therapeutic consistency. Addressing these issues will be essential for future clinical translation.

Overall, this study provides a novel theoretical framework and therapeutic strategy for the treatment of SLE, deepening our understanding of disease mechanisms and therapeutic targets. Notably, the targeted delivery of anti-BAFF mRNA via engineered exosomes offers a promising avenue for precision therapy. With further optimization, this approach has the potential to provide safer and more effective treatment options for patients with SLE and to alleviate the long-term disease burden on individuals and healthcare systems.

In summary, we designed a novel exosome targeting $CD19^+$ cells for delivering anti-BAFF mRNA. It effectively targeted $CD19^+$ B cells, enhanced mRNA half-life, specifically secreted BAFF antibodies in B cell-enriched regions, and exhibited excellent biocompatibility, providing a potential strategy for SLE cell therapy.

MATERIALS AND METHODS

Cells and plasmids

HEK293T cells were cultured in Dulbecco's modified Eagle's medium (Thermo Fisher Scientific, USA) containing 10% fetal bovine serum (Gibco, USA) and 1% penicillin-streptomycin (Invitrogen) at 37°C in a 5% CO_2 atmosphere. *Stbl3*- and *DH5α*-competent cells were used for plasmid transformation.

The pHBLV-CMV-MCS-EF1-ZsGreen-T2A-puro vector (Hanbio Biotechnology, China) and the pLV-EF1α-IRES-puro vector (catalog no. 200526, BGI, China) were used as expression plasmids, while psPAX2 (catalog no. 12260, Addgene) and pMD2.G (catalog no. 12259, Addgene) served as auxiliary plasmids for lentiviral packaging.

Vector construction and preparation of engineered cells

The 1D3 nucleotide sequence encoding the anti-CD19 protein was derived from a patent (no. CN 111601817 A). The gene sequence was synthesized with a CD8α signal peptide fused to the N terminus of the anti-CD19 scFv, followed by a Myc tag and transmembrane domain. The synthesized 1D3 sequence was inserted into the multiple cloning site of the pHBLV-CMV-MCS-EF1-ZsGreen-T2A-puro vector to generate the corresponding lentiviral expression plasmid pLV-1D3. Next, pLV-1D3 was co-transfected into HEK293T cells together with two helper plasmids (psPAX2 and pMD2.G) at a ratio of 4:3:1. After 48 h, lentiviral particles (LV-1D3) were collected and concentrated using PEG8000. Subsequently, HEK293T cells were transduced with LV-1D3, and stable cell lines were selected using puromycin to generate 293T cells stably expressing anti-CD19.

The pLV-EF1α-IRES-puro vector was used to construct plasmids encoding CD63-L7Ae and the anti-$BAFF^{mRNA}$ sequence. The CD63-L7Ae sequence encoded the transmembrane protein CD63 (GenBank: CT010179.1) fused with the RNA-binding protein L7Ae[54] to facilitate mRNA loading into exosomes. The anti-$BAFF^{mRNA}$ sequence, derived from the mRNA of anti-BAFF scFv (patent no. CN 108064250 A) and C/Dbox structure (derived from patent no. CN 114591989 A) was synthesized with a CD8α signal peptide and an FLAG tag. These sequences were separately inserted into the pLV-EF1α-IRES-puro vector to generate pLV-CD63-L7Ae and pLV-anti-BAFF-C/Dbox plasmids. As described above, plasmids were co-transfected into 293T cells, and LVs were collected and concentrated using PEG8000. Subsequently, 293T cells and 1D3 stable cell lines were transduced to generate two engineered cell lines: anti-$BAFF^{mRNA}$ stable cell lines expressing anti-BAFF mRNA and the CD19@Anti-$BAFF^{mRNA}$ stable cell lines expressing both anti-CD19 and anti-BAFF mRNA.

Preparation of exosomes

Exosomes were isolated from culture supernatants by differential centrifugation. Briefly, the culture medium underwent differential centrifugation (800 × *g* for 5 min; 1,200 × *g* for 20 min; 10,000 × *g* for 30 min) to remove cells and debris. The supernatant was then filtered through a 0.22-μm filter and ultracentrifuged at 100,000 × *g* for 1 h to pellet exosomes. The exosome pellet was washed with PBS and re-collected by UC at 100,000 × *g* for 1 h. All centrifugation steps were performed at 4°C, and the final exosome pellets were stored at −80°C until further use. Exosome purity was assessed based on the particle-to-protein ratio, in accordance with MISEV2023 guidelines.[26] Protein concentration of exosomes (mg/mL) was measured using a bicinchoninic acid (BCA) protein assay kit (Beyotime, China), and particle concentration (particle/mL) was determined by NTA.

Exosome characterization

The morphology of free exosomes and CD19@Anti-$BAFF^{mRNA}$ exosomes was examined by TEM (JEM-1400 PLUS, Japan). Briefly,

Figure 6. CD19@Anti-$BAFF^{mRNA}$ Exo alleviated the symptoms of MRL/Lpr mice

(A) Schematic diagram depicting the experimental design for evaluating the efficacy of exosomes from each group in treating MRL/Lpr mice. MRL/Mpj mice were designated as the control group, while MRL/Lpr mice were divided into four groups and treated with exosomes with different modifications (G1: free Exo, G2: CD19 Exo, G3: anti-$BAFF^{mRNA}$ Exo, and G4: CD19@Anti-$BAFF^{mRNA}$ Exo) and tabalumab (G5). Exosomes in each group were administered via the tail vein twice per week, while tabalumab was administered once every 2 weeks. (B) Survival status was recorded daily, and survival curves were analyzed by the log rank test ($n = 10$ per group). (C–E) The levels of dsDNA and ANA in serum (C), urinary protein (D), and cytokines (IL-6, TNF-α, and IFN-γ) in serum (E) were quantified by ELISA. (F and G) Representative images of spleens (F) or lymph nodes (G) from each group and statistical analysis of their weights. (H) Representative images of H&E-stained and Masson-stained kidney sections from each group. Scale bar: 20 μm. Kidney pathology was assessed by GN scores. (I) Representative images of C3 (red) and IgG (green) deposition in the kidneys (top). Scale bar: 50 μm. MFI of C3 and IgG deposition in the kidneys (bottom). Data are presented as the mean ± SEM of three biological replicates. *$p < 0.05$; **$p < 0.01$; ***$p < 0.005$; ns, not significant.

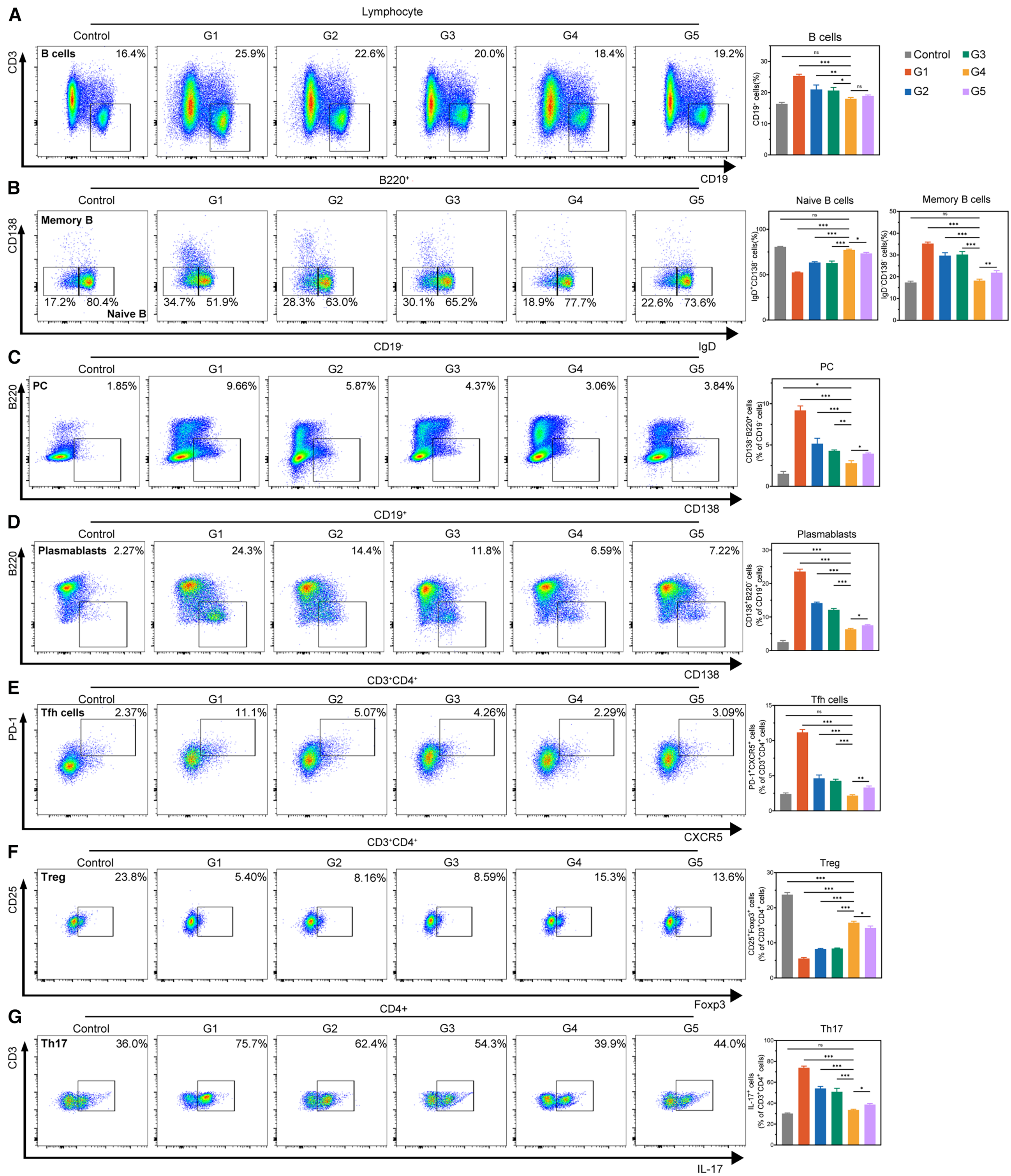

Figure 7. The phenotype of immune cells in MRL/Lpr mice after CD19@Anti-BAFF^{mRNA} Exo treatment

MRL/Mpj mice were designated as the control group, while MRL/Lpr mice were divided into four groups and treated with exosomes with different modifications (G1: free Exo, G2: CD19 Exo, G3: anti-BAFF^{mRNA} Exo, and G4:CD19@Anti-BAFF^{mRNA} Exo) and tabalumab (G5). The expression of cell-specific surface markers was detected by flow

(legend continued on next page)

10 μL exosomes were loaded onto a 300-mesh copper mesh, followed by negative staining using 2% aqueous uranyl acetate for 2 min at room temperature; excess solution was removed by suctioning with filter paper. After air-drying, the samples were imaged using TEM.

The size distribution and concentration of exosomes were analyzed by tracking Brownian motion using a NanoSight NS300 system (NanoSight NS300, Malvern, UK). TExosome suspensions were diluted in PBS and injected into the NanoSight sample chamber. The samples were continuously analyzed via the top plate of the flow cell at a steady speed, and the Brownian motion of nanoparticles was recorded three times (42 s each turn). The measured data were analyzed with the use of NTA 3.0 analysis software (Malvern). The hydrodynamic size and surface zeta potential of the exosomes were measured using a Zetasizer (Malvern).

Western blot analysis

Cells and exosomes were treated with radioimmunoprecipitation assay buffer (0.5% NP-40, 150 mM NaCl, 50 mM Tris-HCl, 5 mM $Na_2EDTA \cdot 2H_2O$, 1 mM PMSF). Protein concentrations were determined using a BCA Protein Assay Kit (Thermo Fisher Scientific). Protein samples (20 μg/well) were loaded onto 10% SDS-PAGE gels and separated. Following the transfer, the membranes were blocked with 5% skim milk or BSA for 1 h at room temperature, washed twice with PBS-T, and incubated overnight at 4°C three times with corresponding primary antibodies. After washing, membranes were incubated with corresponding horseradish peroxidase (HRP)-coupled secondary antibodies (Cell Signaling Technology [CST], USA) for 2 h at room temperature. Protein bands were visualized using an ECL detection system (GE Healthcare, USA) on a MiniMedical/90 imaging system (Image Works, USA). The primary antibodies used included calnexin (catalog no. ab133615, Abcam, UK), CD81 (catalog no. ab109201, Abcam), CD63 (catalog no. ab134045, Abcam), Hsp70 (catalog no. ab181606, Abcam), TSG101 (catalog no. ab125011, Abcam), glyceraldehyde-3-phosphate dehydrogenase (GAPDH, catalog no. ab181602, Abcam), β-actin (catalog no. 4970S, CST), FLAG-Tag (DYKDDDDK-Tag, catalog no. 14793S, CST), and Myc-Tag (catalog no. 3946S, CST).

RT-qPCR analysis

The extraction of exosomal RNA was performed via TRIzol reagent (Invitrogen, USA), and RT-qPCR was conducted on a MyiQcycler (Bio-Rad, USA) via SYBR Green I (Molecular Probes, USA). The mRNA levels of the target gene were normalized to those of mouse GAPDH mRNA. The following primer sequences were used: forward: 5′-AGACTCAAGCCCGGTGAAAG-3′; reverse: 5′-GGCT TGTCGTACCCAGTTGA-3′.

Laser confocal microscopy

To examine the targeting of B lymphocytes by CD19@Anti-$BAFF^{mRNA}$ Exo, both components were incubated together. Exosomes were stained with DiO (catalog no. C1038, Beyotime) at 37°C for 20 min, purified by UC to remove free dye, and the labeled exosome was suspended in 100 μL PBS after washing twice. Then, the exosomes were incubated with B lymphocytes in a confocal dish for 4 h at 37°C. After washing three times with PBS, cells were stained with DiI (catalog no. C1991S, Beyotime) for 20 min at 37°C. After additional washing three times with PBS, cells were imaged using a confocal laser scanning microscope (Zeiss LSM 880). Fluorescence was detected using the following excitation/emission wavelengths: DiO ([excitation wavelength/emission wavelength] ex 488 nm/em 530 nm) and DiI (ex 549 nm/em 565 nm).

Biodistribution of exosomes

Exosomes were labeled with DiR (catalog no. M5122, AbMole BioScience, USA) by incubation at 37°C for 30 min, followed by UC at 100,000 × *g* for 60 min to remove free DiR, and DiR fluorescent dye-labeled exosomes were obtained and diluted in PBS. The mice were treated with different groups of exosomes by intravenous injection at the same dose of 10 mg/kg and euthanized after 24 h. The Xenogen IVIS Spectrum system (Caliper Life Sciences, USA) was used to detect the biodistribution of exosomes in different organs (heart, lungs, liver, spleen, stomach, intestines, and kidneys) of the mice.

Antibody production efficiency of exosome treatment

ELISA analysis was used to detect the production efficiency of anti-BAFF antibodies in cell supernatants and serum. Briefly, BAFF was added to the microplate as an antigen and incubated overnight at 4°C to coat the microplate with BAFF. Following a washing step, standards and samples were added in gradients. After incubation at 37°C for 1 h and subsequent washing, an HRP-conjugated antibody was incorporated. After another 1-h incubation at 37°C and a subsequent washing step, a chromogenic solution and a stop solution were added. Finally, the absorbance was measured using a microplate reader to determine the amount of anti-BAFF antibodies in the samples. A Mouse FLAG tag ELISA Kit (catalog no. MM-46037M1, MEIMIAN, China) was also used to indirectly reflect antibody production efficiency.

Activation of B cells

Mouse spleen cells were stimulated with BAFF (10 μg/mL) and co-cultured with exosomes for 48 h. The mRNA levels of IL-1β, IL-6, TNF-α, IL-10, TGF-β, IL-4, and IL-12p40 were normalized to those of β-actin mRNA. The sequences of the primers used in this study are listed in Table S1. The mean fluorescence intensity

cytometry. Representative flow cytometry plots and statistical analysis of the proportion of B cells ($CD3^-CD19^+$) (A), naive B cells ($B220^+IgD^+CD138^-$), memory B cells ($B220^+IgD^-CD138^-$) (B), plasma cells ($CD19^-B220^-CD138^+$) (C), plasmablasts ($CD19^+B220^-CD138^+$) (D), Tfh cells ($CD3^+CD4^+PD\text{-}1^+CXCR5^+$) (E), Treg cells ($CD3^-CD4^+CD25^+Foxp3^+$) (F), and Th17 cells ($CD3^+CD4^+IL\text{-}17^+$) (G) in spleens. Data are presented as the mean ± SEM of three biological replicates. **p* < 0.05; ***p* < 0.01; ****p* < 0.005; ns, not significant.

(MFI) of MHC class II, CD69, and CD80 in $CD19^+$ B cells was detected by flow cytometry.

Proliferation of B cells

Mouse spleen cells stained with CFSE were stimulated with BAFF (10 μg/mL) and co-cultured with exosomes for 72 h. Flow cytometry was used to detect the proportion of proliferating B cells.

Animals

The animal experiments were approved and carried out in accordance with the protocols approved by the Animal Care and Use Committee of the Fifth Affiliated Hospital of Sun Yat-sen University. Healthy C57BL/6J mice (5–6 weeks old) and MRL/Lpr mice (12 weeks old) were obtained from the Guangdong Medical Experimental Animal Center.

Lupus mice induced by ALD-DNA

To obtain lupus mice induced by ALD-DNA, mouse splenocytes were first cultured in RPMI 1640 medium (Thermo Fisher Scientific) containing 5 μg/mL ConA (catalog no. L7647, Sigma-Aldrich, USA) at 37°C in a 5% CO_2 environment. After 6 days, viable cells were collected to extract ALD-DNA. The ALD-DNA was thoroughly mixed with adjuvants (complete Freund's adjuvant [catalog no. F5881, Sigma-Aldrich] in week 0 and incomplete Freund's adjuvant [catalog no. F5506, Sigma-Aldrich] in weeks 2 and 4), and 100 μL of the mixture (containing 50 μg ALD-DNA) was subcutaneously injected into the back of each mouse.[55] After three subcutaneous injections were completed, the mice were randomly divided into four groups for subsequent treatment.

Analysis of the therapeutic effect on lupus mice

Spleens and lymph nodes were removed, and their sizes and weights were measured to assess changes in lymphatic organs. Kidneys were used for histological analysis. Spleens were also used for flow cytometry analysis. To analyze cytokines in peripheral blood, eyeball blood samples were collected and centrifuged at 3,000 $\times$ *g* for 5 min after 30 min of coagulation. The serum was then collected and used for ELISAs, including the Mouse dsDNA ELISA Kit (catalog no. MM-46273M1, MEIMIAN) and the Mouse Urinary Protein (UP) ELISA Kit (catalog no. MM-44287M1, MEIMIAN).

Flow cytometry

To analyze splenic immune cells, spleen tissue was carefully ground and filtered through a 100-mesh nylon mesh into a centrifuge tube. Following centrifugation at 1,800 rpm for 5 min, red blood cells in the sediment were lysed using lysis buffer at room temperature for 5–10 min. After further filtration, a single-cell suspension was obtained and stained with the corresponding fluorescent antibodies for flow cytometric analysis. The antibodies used were as follows: BV421-anti-mouse CD3 (BioLegend, clone 17A2), allophycocyanin (APC)-anti-mouse CD19 (BioLegend, clone 1D3), fluorescein isothiocyanate (FITC)-anti-mouse B220 (BioLegend, clone RA36B2), phycoerythrin (PE)-anti-mouse CD138 (BioLegend, clone 281-2), FITC-anti-mouse IgD (BioLegend, clone 11-26c.2a), FITC-anti-mouse CD3 (BioLegend, clone 17A2), APC-anti-mouse CD4 (BioLegend, clone GK1.5), PE/cyanine7-anti-mouse PD-1 (BioLegend, clone 29F.1A12), V450-anti-mouse CXCR5 (BioLegend, clone L138D7), APC-anti-mouse Ly6C (BioLegend, clone HK1.4), PE/cyanine7-anti-mouse Ly6G (BioLegend, clone 1A8), FITC-anti-mouse CD11c (BioLegend, clone N418), V450-anti-mouse F4/80 (BioLegend, clone BM8), PE/cyanine7-anti-mouse CD25 (BioLegend, clone PC-61.5.3), V450-anti-mouse Foxp3 (BioLegend, clone 3G3), and BV421-anti-mouse IL-17 (BioLegend, clone 17F3). The corresponding IgG antibody was used as an isotype control.

Histologic analysis

Kidneys from each group were removed, bisected, and fixed in formalin. Paraffin embedding, sectioning, and H&E staining, as well as Masson staining, were performed by Taize Biotechnology. Sections were observed using a slide scanner system (3DHistech, P250FLASH, Hungary). GN was scored by a pathologist in a blinded manner on a scale of 1–6: 1, normal kidney; 2, mesangial expansion and increased mesangial cellularity with patent capillary loops; 3, enlarged glomeruli with moderate endocapillary cellularity; 4, three characteristics with marked endocapillary hypercellularity and loss of patency of most capillary loops; 5, few glomeruli with necrosis (karyorrhexis) or few active (cellular or fibrocellular) or organized (fibrous) crescents, necrosis (karyorrhexis), obliteration of glomerular architecture, and segmental/global sclerosis; 6, many active (cellular or fibrocellular) or organized (fibrous) crescents, necrosis (karyorrhexis), obliteration of glomerular architecture, and segmental/global sclerosis.[56]

Immunofluorescence analysis

To detect the deposition of IgG and complement C3, the kidneys were embedded with Optimal Cutting Temperature compound (SAKURA, Japan), frozen at −80°C, and sectioned. The sections were permeabilized with Triton X-100. After thorough washing, the samples were incubated with 5% BSA for blocking to reduce specific binding. Primary antibodies (Complement C3 Polyclonal Antibody, Thermo Fisher) were applied and incubated overnight at 4°C in a humid chamber. The primary antibody was washed with PBS. Subsequently, the fluorescently labeled secondary antibody rabbit anti-mouse IgG (heavy and light chains [H&L]) Alexa Fluor 488 (catalog no. A11059, Thermo Fisher) and goat anti-rat IgG H&L Alexa Fluor 594 (ab150160, Abcam) were added, followed by incubation at room temperature for 1 h in a humid chamber. After another washing step, the cell nuclei were stained with DAPI (catalog no. C1005, Beyotime). Finally, the samples were mounted with an anti-fluorescence quenching mounting medium and observed using a confocal microscope (Zeiss LSM 880).

In vivo safety analysis

The mice were divided into four groups: free Exo, CD19 Exo, anti-$BAFF^{mRNA}$ Exo, and CD19@Anti-$BAFF^{mRNA}$ Exo. All agents were administered intravenously at the same dose (10 mg/kg). The mice were sacrificed on day 14, and major organs (the lungs, liver, spleen, heart, and kidneys) were fixed in 4% paraformaldehyde buffer

solution for H&E staining and histologic analysis. Serum was used to detect changes in biochemical indicators.

Statistical analysis

All experiments were conducted with at least three independent biological replicates, and data are presented as mean ± SEM. Data analyses were performed using GraphPad Prism 10.1.1 software (GraphPad, USA). Two-tailed Student's *t* tests were used to compare two groups, whereas one-way analysis of variance followed by Tukey's post-test was used for comparing more than two groups. The statistical significance was set as $^{*}p < 0.05$, $^{**}p < 0.01$, and $^{***}p < 0.005$ (ns, not significant).

DATA AND CODE AVAILABILITY

The authors confirm that materials and protocols will be made available on request, subject to appropriate agreements.

ACKNOWLEDGMENTS

The study was supported by the National Natural Science Foundation of China (grant no. 82300809 to J.S., grant no. 82072062 to X.H., grant no. 82270016 to Y.W.), the Natural Science Foundation of Guangdong Province (grant no. 2023A1515030065 to Y.W.), and the Youth S&T Talent Support Programme of Guangdong Provincial Association for Science and Technology (grant no. SKXRC2025142 to J.S.).

AUTHOR CONTRIBUTIONS

L.Z. conceived the study; designed, performed, and interpreted the experiments; prepared the figures; and wrote the manuscript. J.S. designed, performed, and interpreted the experiments and reviewed the manuscript. S.Z. and X.D provided the resources and analyzed the data. X.H. and Y.W., as the corresponding authors, coordinated the research process, supervised the overall project, corresponded with the journal and reviewers, and finalized the manuscript.

DECLARATION OF INTERESTS

The authors declare no competing interests.

SUPPLEMENTAL INFORMATION

Supplemental information can be found online at https://doi.org/10.1016/j.ymthe.2026.01.037.

REFERENCES

1. Thanou, A., Jupe, E., Purushothaman, M., Niewold, T.B., and Munroe, M.E. (2021). Clinical disease activity and flare in SLE: Current concepts and novel biomarkers. J. Autoimmun. *119*, 102615. https://doi.org/10.1016/j.jaut.2021.102615.

2. Li, M., Zhao, Y., Zhang, Z., Huang, C., Liu, Y., Gu, J., Zhang, X., Xu, H., Li, X., Wu, L., et al. (2020). 2020 Chinese Guidelines for the Diagnosis and Treatment of Systemic Lupus Erythematosus. Rheumatol. Immunol. Res. *1*, 5–23. https://doi.org/10.2478/rir-2020-0009.

3. Tagami, N., Yuda, J., and Goto, Y. (2024). Current status of BAFF targeting immunotherapy in B-cell neoplasm. Int. J. Clin. Oncol. *29*, 1676–1683. https://doi.org/10.1007/s10147-024-02611-2.

4. Karrar, S., and Cunninghame Graham, D.S. (2018). Abnormal B Cell Development in Systemic Lupus Erythematosus: What the Genetics Tell Us. Arthritis Rheumatol. *70*, 496–507. https://doi.org/10.1002/art.40396.

5. Crow, M.K. (2023). Pathogenesis of systemic lupus erythematosus: risks, mechanisms and therapeutic targets. Ann. Rheum. Dis. *82*, 999–1014. https://doi.org/10.1136/ard-2022-223741.

6. Vincent, F.B., Morand, E.F., Schneider, P., and Mackay, F. (2014). The BAFF/APRIL system in SLE pathogenesis. Nat. Rev. Rheumatol. *10*, 365–373. https://doi.org/10.1038/nrrheum.2014.33.

7. Jackson, S.W., and Davidson, A. (2019). BAFF inhibition in SLE-Is tolerance restored? Immunol. Rev. *292*, 102–119. https://doi.org/10.1111/imr.12810.

8. Möckel, T., Basta, F., Weinmann-Menke, J., and Schwarting, A. (2021). B cell activating factor (BAFF): Structure, functions, autoimmunity and clinical implications in Systemic Lupus Erythematosus (SLE). Autoimmun. Rev. *20*, 102736. https://doi.org/10.1016/j.autrev.2020.102736.

9. Smulski, C.R., and Eibel, H. (2018). BAFF and BAFF-Receptor in B Cell Selection and Survival. Front. Immunol. *9*, 2285. https://doi.org/10.3389/fimmu.2018.02285.

10. Yarchoan, M., Ho, W.J., Mohan, A., Shah, Y., Vithayathil, T., Leatherman, J., Dennison, L., Zaidi, N., Ganguly, S., Woolman, S., et al. (2020). Effects of B cell-activating factor on tumor immunity. JCI insight *5*, e136417. https://doi.org/10.1172/jci.insight.136417.

11. Bauer, J.W., Baechler, E.C., Petri, M., Batliwalla, F.M., Crawford, D., Ortmann, W.A., Espe, K.J., Li, W., Patel, D.D., Gregersen, P.K., and Behrens, T.W. (2006). Elevated serum levels of interferon-regulated chemokines are biomarkers for active human systemic lupus erythematosus. PLoS Med. *3*, e491. https://doi.org/10.1371/journal.pmed.0030491.

12. Howe, H.S., Thong, B.Y.H., Kong, K.O., Chng, H.H., Lian, T.Y., Chia, F.L., Tay, K.S.S., Lau, T.C., Law, W.G., Koh, E.T., and Leung, B.P. (2017). Associations of B cell-activating factor (BAFF) and anti-BAFF autoantibodies with disease activity in multi-ethnic Asian systemic lupus erythematosus patients in Singapore. Clin. Exp. Immunol. *189*, 298–303. https://doi.org/10.1111/cei.12975.

13. Navarra, S.V., Guzmán, R.M., Gallacher, A.E., Hall, S., Levy, R.A., Jimenez, R.E., Li, E.K.M., Thomas, M., Kim, H.Y., León, M.G., et al. (2011). Efficacy and safety of belimumab in patients with active systemic lupus erythematosus: a randomised, placebo-controlled, phase 3 trial. Lancet (London, England) *377*, 721–731. https://doi.org/10.1016/s0140-6736(10)61354-2.

14. Furie, R., Rovin, B.H., Houssiau, F., Malvar, A., Teng, Y.K.O., Contreras, G., Amoura, Z., Yu, X., Mok, C.C., Santiago, M.B., et al. (2020). Two-Year, Randomized, Controlled Trial of Belimumab in Lupus Nephritis. N. Engl. J. Med. *383*, 1117–1128. https://doi.org/10.1056/NEJMoa2001180.

15. Zhang, F., Bae, S.C., Bass, D., Chu, M., Egginton, S., Gordon, D., Roth, D.A., Zheng, J., and Tanaka, Y. (2018). A pivotal phase III, randomised, placebo-controlled study of belimumab in patients with systemic lupus erythematosus located in China, Japan and South Korea. Ann. Rheum. Dis. *77*, 355–363. https://doi.org/10.1136/annrheumdis-2017-211631.

16. Van Hoecke, L., and Roose, K. (2019). How mRNA therapeutics are entering the monoclonal antibody field. J. Transl. Med. *17*, 54. https://doi.org/10.1186/s12967-019-1804-8.

17. Vanover, D., Zurla, C., Peck, H.E., Orr-Burks, N., Joo, J.Y., Murray, J., Holladay, N., Hobbs, R.A., Jung, Y., Chaves, L.C.S., et al. (2022). Nebulized mRNA-Encoded Antibodies Protect Hamsters from SARS-CoV-2 Infection. Adv. Sci. *9*, e2202771. https://doi.org/10.1002/advs.202202771.

18. Rybakova, Y., Kowalski, P.S., Huang, Y., Gonzalez, J.T., Heartlein, M.W., DeRosa, F., Delcassian, D., and Anderson, D.G. (2019). mRNA Delivery for Therapeutic Anti-HER2 Antibody Expression In Vivo. Mol. Ther. *27*, 1415–1423. https://doi.org/10.1016/j.ymthe.2019.05.012.

19. Rurik, J.G., Tombácz, I., Yadegari, A., Méndez Fernández, P.O., Shewale, S.V., Li, L., Kimura, T., Soliman, O.Y., Papp, T.E., Tam, Y.K., et al. (2022). CAR T cells produced in vivo to treat cardiac injury. Science (New York, N.Y.) *375*, 91–96. https://doi.org/10.1126/science.abm0594.

20. Wang, C., Pan, C., Yong, H., Wang, F., Bo, T., Zhao, Y., Ma, B., He, W., and Li, M. (2023). Emerging non-viral vectors for gene delivery. J. Nanobiotechnology *21*, 272. https://doi.org/10.1186/s12951-023-02044-5.

21. Liu, Y., Li, D., Liu, Z., Zhou, Y., Chu, D., Li, X., Jiang, X., Hou, D., Chen, X., Chen, Y., et al. (2015). Targeted exosome-mediated delivery of opioid receptor Mu siRNA for the treatment of morphine relapse. Sci. Rep. *5*, 17543. https://doi.org/10.1038/srep17543.

22. Yang, Z., Shi, J., Xie, J., Wang, Y., Sun, J., Liu, T., Zhao, Y., Zhao, X., Wang, X., Ma, Y., et al. (2020). Large-scale generation of functional mRNA-encapsulating exosomes via cellular nanoporation. Nat. Biomed. Eng. *4*, 69–83. https://doi.org/10.1038/s41551-019-0485-1.

23. Kojima, R., Bojar, D., Rizzi, G., Hamri, G.C.E., El-Baba, M.D., Saxena, P., Ausländer, S., Tan, K.R., and Fussenegger, M. (2018). Designer exosomes produced by implanted cells intracerebrally deliver therapeutic cargo for Parkinson's disease treatment. Nat. Commun. *9*, 1305. https://doi.org/10.1038/s41467-018-03733-8.

24. Krishnan, V., Xu, X., Kelly, D., Snook, A., Waldman, S.A., Mason, R.W., Jia, X., and Rajasekaran, A.K. (2015). CD19-Targeted Nanodelivery of Doxorubicin Enhances Therapeutic Efficacy in B-Cell Acute Lymphoblastic Leukemia. Mol. Pharm. *12*, 2101–2111. https://doi.org/10.1021/acs.molpharmaceut.5b00071.

25. Mackensen, A., Müller, F., Mougiakakos, D., Böltz, S., Wilhelm, A., Aigner, M., Völkl, S., Simon, D., Kleyer, A., Munoz, L., et al. (2022). Anti-CD19 CAR T cell therapy for refractory systemic lupus erythematosus. Nat. Med. *28*, 2124–2132. https://doi.org/10.1038/s41591-022-02017-5.

26. Welsh, J.A., Goberdhan, D.C.I., O'Driscoll, L., Buzas, E.I., Blenkiron, C., Bussolati, B., Cai, H., Di Vizio, D., Driedonks, T.A.P., Erdbrügger, U., et al. (2024). Minimal information for studies of extracellular vesicles (MISEV2023): From basic to advanced approaches. J. Extracell. Vesicles *13*, e12404. https://doi.org/10.1002/jev2.12404.

27. Siegel, C.H., and Sammaritano, L.R. (2024). Systemic Lupus Erythematosus: A Review. Jama *331*, 1480–1491. https://doi.org/10.1001/jama.2024.2315.

28. Singh, J.A., Shah, N.P., and Mudano, A.S. (2021). Belimumab for systemic lupus erythematosus. Cochrane Database Syst. Rev. *2*, Cd010668. https://doi.org/10.1002/14651858.CD010668.pub2.

29. Tani, C., Zucchi, D., Cardelli, C., Elefante, E., Signorini, V., Schilirò, D., Cascarano, G., Gualtieri, L., Valevich, A., Puccetti, G., et al. (2024). Analysis of belimumab prescription and outcomes in a 10-year monocentric cohort: is there an advantage with early use? RMD open *10*, e003981. https://doi.org/10.1136/rmdopen-2023-003981.

30. Kojima, K., Ichinose, K., Umeda, M., Shimizu, T., Sato, S., Suzuki, T., Nakashima, Y., Okada, A., Horai, Y., Fujikawa, K., et al. (2025). Enhancing systemic lupus erythematosus treatment outcomes with an early initiation of belimumab: insights from a multicenter retrospective study within the first five years. Arthritis Res. Ther. *27*, 116. https://doi.org/10.1186/s13075-025-03581-0.

31. Hoi, A., Igel, T., Mok, C.C., and Arnaud, L. (2024). Systemic lupus erythematosus. Lancet (London, England) *403*, 2326–2338. https://doi.org/10.1016/s0140-6736(24)00398-2.

32. You, Y., Tian, Y., Guo, R., Shi, J., Kwak, K.J., Tong, Y., Estania, A.P., Hsu, W.H., Liu, Y., Hu, S., et al. (2025). Extracellular vesicle-mediated VEGF-A mRNA delivery rescues ischaemic injury with low immunogenicity. Eur. Heart J. *46*, 1662–1676. https://doi.org/10.1093/eurheartj/ehae883.

33. Chen, Y., Lei, X., Xu, J., Chen, X., Pan, H., Zhang, Q., Wang, J., Ren, P., Lan, L., Shi, N., et al. (2025). Belimumab versus telitacicept in sequential treatment after rituximab for refractory lupus nephritis: a real-world multicentre study. Lupus Sci. Med. *12*, e001296. https://doi.org/10.1136/lupus-2024-001296.

34. Guo, C., Tang, Y., Zeng, L., You, X., Luo, S., Du, Y., Wang, L., Wang, L., Wang, J., Chen, J., and Zhou, Y. (2025). Therapeutic Potential of Lipid Nanoparticle-Encapsulated CD19-Targeting mRNAs in Lupus and Rheumatoid Arthritis. Adv. Sci. *12*, e2501628. https://doi.org/10.1002/advs.202501628.

35. Gallagher, S., Yusuf, I., McCaughtry, T.M., Turman, S., Sun, H., Kolbeck, R., Herbst, R., and Wang, Y. (2016). MEDI-551 Treatment Effectively Depletes B Cells and Reduces Serum Titers of Autoantibodies in Mice Transgenic for Sle1 and Human CD19. Arthritis Rheumatol. *68*, 965–976. https://doi.org/10.1002/art.39503.

36. Wang, L., Yin, H., Jiang, J., Li, Q., Gao, C., Li, W., Zhang, B., Xin, Y., Li, H., Zhao, M., and Lu, Q. (2024). A rationally designed CD19 monoclonal antibody-triptolide conjugate for the treatment of systemic lupus erythematosus. Acta Pharm. Sin. B *14*, 4560–4576. https://doi.org/10.1016/j.apsb.2024.06.024.

37. Jiang, T., Jing, S., Li, H., Xiao, B., Jin, J., Sun, X., Wang, J., Liang, J., Cai, T., Hu, H., et al. (2025). Localized Delivery of the mRNAs Encoding CD47 Inhibitor and Interleukins 12, 15, and 21 Elicits Robust Antitumor Immunity. Adv. Sci. *12*, e17205. https://doi.org/10.1002/advs.202417205.

38. Tai, W., Yang, K., Liu, Y., Li, R., Feng, S., Chai, B., Zhuang, X., Qi, S., Shi, H., Liu, Z., et al. (2023). A lung-selective delivery of mRNA encoding broadly neutralizing antibody against SARS-CoV-2 infection. Nat. Commun. *14*, 8042. https://doi.org/10.1038/s41467-023-43798-8.

39. Jin, X., Xu, Q., Pu, C., Zhu, K., Lu, C., Jiang, Y., Xiao, L., Han, Y., and Lu, L. (2021). Therapeutic efficacy of anti-CD19 CAR-T cells in a mouse model of systemic lupus erythematosus. Cell. Mol. Immunol. *18*, 1896–1903. https://doi.org/10.1038/s41423-020-0472-1.

40. Feng, J., Huo, D., Hong, R., Jin, X., Cao, H., Shao, M., Wen, R., Zhang, Q., Zhang, M., Fu, S., et al. (2025). Co-infusion of CD19-targeting and BCMA-targeting CAR-T cells for treatment-refractory systemic lupus erythematosus: a phase 1 trial. Nat. Med. *31*, 3725–3736. https://doi.org/10.1038/s41591-025-03937-8.

41. Schett, G., Mackensen, A., and Mougiakakos, D. (2023). CAR T-cell therapy in autoimmune diseases. Lancet (London, England) *402*, 2034–2044. https://doi.org/10.1016/s0140-6736(23)01126-1.

42. Li, M., Huang, H., Wei, X., Li, H., Li, J., Xie, B., Yang, Y., Fang, X., Wang, L., Zhang, X., et al. (2025). Clinical investigation on nebulized human umbilical cord MSC-derived extracellular vesicles for pulmonary fibrosis treatment. Signal Transduct. Target. Ther. *10*, 179. https://doi.org/10.1038/s41392-025-02262-3.

43. Hadizadeh, A., Akbari Asbagh, R., Heirani-Tabasi, A., Soleimani, M., Gorovanchi, P., Ebrahimi Daryani, N., Vahedi, A., Nazari, H., Banikarimi, S.P., Abbaszade Dibavar, M., et al. (2024). Localized Administration of Mesenchymal Stem Cell-Derived Exosomes for the Treatment of Refractory Perianal Fistula in Patients With Crohn's Disease: A Phase II Clinical Trial. Dis. Colon Rectum *67*, 1564–1575. https://doi.org/10.1097/dcr.0000000000003502.

44. Xu, Z., Zeng, S., Gong, Z., and Yan, Y. (2020). Exosome-based immunotherapy: a promising approach for cancer treatment. Mol. Cancer *19*, 160. https://doi.org/10.1186/s12943-020-01278-3.

45. Zeng, L., Yang, K., Wu, Y., Yu, G., Yan, Y., Hao, M., Song, T., Li, Y., Chen, J., and Sun, L. (2024). Telitacicept: A novel horizon in targeting autoimmunity and rheumatic diseases. J. Autoimmun. *148*, 103291. https://doi.org/10.1016/j.jaut.2024.103291.

46. Budde, L.E., Sehn, L.H., Matasar, M., Schuster, S.J., Assouline, S., Giri, P., Kuruvilla, J., Canales, M., Dietrich, S., Fay, K., et al. (2022). Safety and efficacy of mosunetuzumab, a bispecific antibody, in patients with relapsed or refractory follicular lymphoma: a single-arm, multicentre, phase 2 study. Lancet Oncol. *23*, 1055–1065. https://doi.org/10.1016/s1470-2045(22)00335-7.

47. Hsu, F.F., Liang, K.H., Kumari, M., Chen, W.Y., Lin, H.T., Cheng, C.M., Tao, M.H., and Wu, H.C. (2022). An efficient approach for SARS-CoV-2 monoclonal antibody production via modified mRNA-LNP immunization. Int. J. Pharm. *627*, 122256. https://doi.org/10.1016/j.ijpharm.2022.122256.

48. Suurs, F.V., Lub-de Hooge, M.N., de Vries, E.G.E., and de Groot, D.J.A. (2019). A review of bispecific antibodies and antibody constructs in oncology and clinical challenges. Pharmacol. Ther. *201*, 103–119. https://doi.org/10.1016/j.pharmthera.2019.04.006.

49. Pardi, N., Secreto, A.J., Shan, X., Debonera, F., Glover, J., Yi, Y., Muramatsu, H., Ni, H., Mui, B.L., Tam, Y.K., et al. (2017). Administration of nucleoside-modified mRNA encoding broadly neutralizing antibody protects humanized mice from HIV-1 challenge. Nat. Commun. *8*, 14630. https://doi.org/10.1038/ncomms14630.

50. Huang, H., and Wei, X. (2024). Therapeutic potential of CD20/CD3 bispecific antibodies in the treatment of autoimmune diseases. Rheumatol. Immunol. Res. *5*, 209–216. https://doi.org/10.1515/rir-2024-0029.

51. Markouli, M., Ullah, F., Unlu, S., Omar, N., Lopetegui-Lia, N., Duco, M., Anwer, F., Raza, S., and Dima, D. (2023). Toxicity Profile of Chimeric Antigen Receptor T-Cell and Bispecific Antibody Therapies in Multiple Myeloma: Pathogenesis, Prevention and Management. Curr. Oncol. *30*, 6330–6352. https://doi.org/10.3390/curroncol30070467.

52. Herrera, M., Pretelli, G., Desai, J., Garralda, E., Siu, L.L., Steiner, T.M., and Au, L. (2024). Bispecific antibodies: advancing precision oncology. Trends Cancer *10*, 893–919. https://doi.org/10.1016/j.trecan.2024.07.002.

53. Han, X., Alameh, M.G., Butowska, K., Knox, J.J., Lundgreen, K., Ghattas, M., Gong, N., Xue, L., Xu, Y., Lavertu, M., et al. (2023). Adjuvant

lipidoid-substituted lipid nanoparticles augment the immunogenicity of SARS-CoV-2 mRNA vaccines. Nat. Nanotechnol. *18*, 1105–1114. https://doi.org/10.1038/s41565-023-01404-4.

54. Moore, T., Zhang, Y., Fenley, M.O., and Li, H. (2004). Molecular basis of box C/D RNA-protein interactions; cocrystal structure of archaeal L7Ae and a box C/D RNA. Structure (London, England : 1993) *12*, 807–818. https://doi.org/10.1016/j.str.2004.02.033.

55. Qiao, B., Wu, J., Chu, Y.W., Wang, Y., Wang, D.P., Wu, H.S., and Xiong, S.D. (2005). Induction of systemic lupus erythematosus-like syndrome in syngeneic mice by immunization with activated lymphocyte-derived DNA. Rheumatology (Oxford, England) *44*, 1108–1114. https://doi.org/10.1093/rheumatology/keh656.

56. Leibler, C., John, S., Elsner, R.A., Thomas, K.B., Smita, S., Joachim, S., Levack, R.C., Callahan, D.J., Gordon, R.A., Bastacky, S., et al. (2022). Genetic dissection of TLR9 reveals complex regulatory and cryptic proinflammatory roles in mouse lupus. Nat. Immunol. *23*, 1457–1469. https://doi.org/10.1038/s41590-022-01310-2.

YMTHE, Volume *34*

Supplemental Information

Anti-CD19-engineered exosomes enable B cell-targeted anti-BAFF mRNA delivery to alleviate lupus progression

Linda Zeng, Jingxian Shu, Shuping Zhong, Xin Dong, Yongjian Wu, and Xi Huang

Table S1 Sequences of primers

Name of primers	Sequences of primers
Mouse-β-actin	Forward: GATTACTGCTCTGGCTCCTAGC<br>Reverse: GACTCATCGTACTCCTGCTTGC
Mouse-GAPDH	Forward: GGAGAGTGTTTCCTCGTCCC<br>Reverse: ATGAAGGGGTCGTTGATGGC
Mouse-IL-4	Forward: ATCATCGGCATTTTGAACGAGGTC<br>Reverse: ACCTTGGAAGCCCTACAGACGA
Mouse-IL-10	Forward: CGGGAAGACAATAACTGCACCC<br>Reverse: CGGTTAGCAGTATGTTGTCCAGC
Mouse-TNF-α	Forward: GGTGCCTATGTCTCAGCCTCTT<br>Reverse: GCCATAGAACTGATGAGAGGGAG
Mouse-IL-1β	Forward: TGGACCTTCCAGGATGAGGACA<br>Reverse: GTTCATCTCGGAGCCTGTAGTG
Mouse-TGF-β	Forward: TGATACGCCTGAGTGGCTGTCT<br>Reverse: CACAAGAGCAGTGAGCGCTGAA
Mouse-IL-6	Forward: TACCACTTCACAAGTCGGAGGC<br>Reverse: CTGCAAGTGCATCATCGTTGTTC
Mouse-IL-12p40	Forward: TCACGCTACCTCCTCTTT<br>Reverse: GGTTTCGGGACTGGCTAA

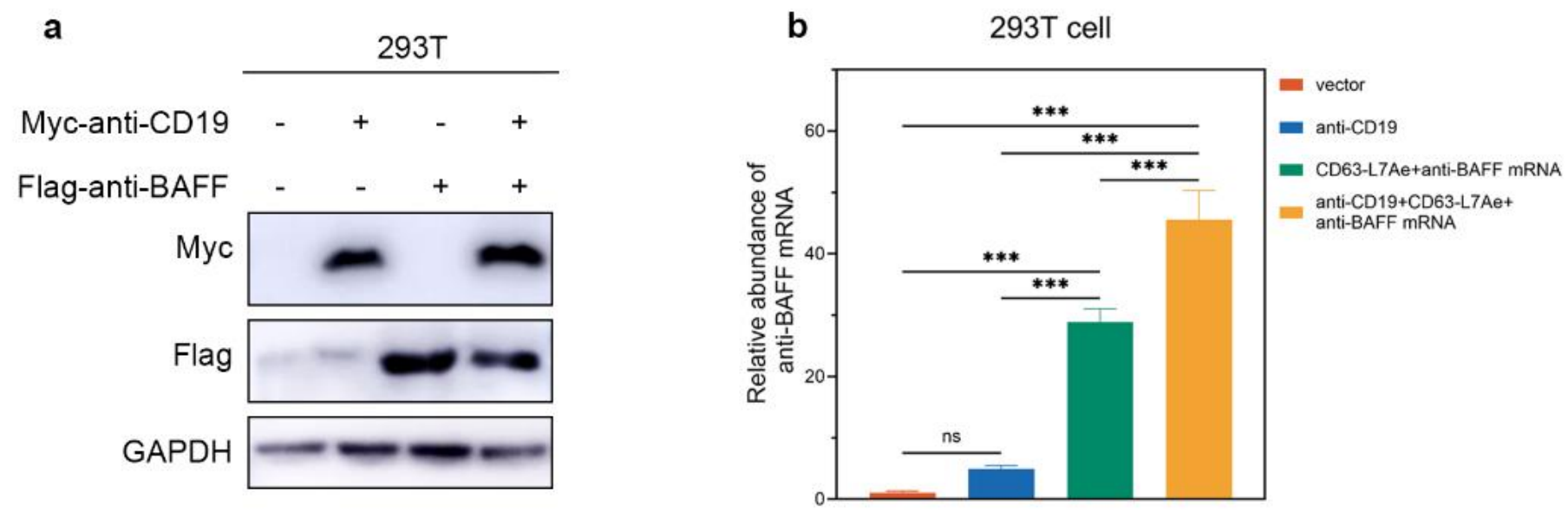

Figure S1 Characterization of stable transfected HEK293T cell line.

(a) The expression of Myc-anti-CD19 and Flag-anti-BAFF in each stable cell line were analyzed by Western blotting. **(b)** The relative abundance of anti-BAFF mRNA in each stable cell line was analyzed by quantitative real-time PCR. Data are presented as the mean±SEM of three biological replicates. *$p < 0.05$, **$p < 0.01$, ***$p < 0.005$, ns: not significant.

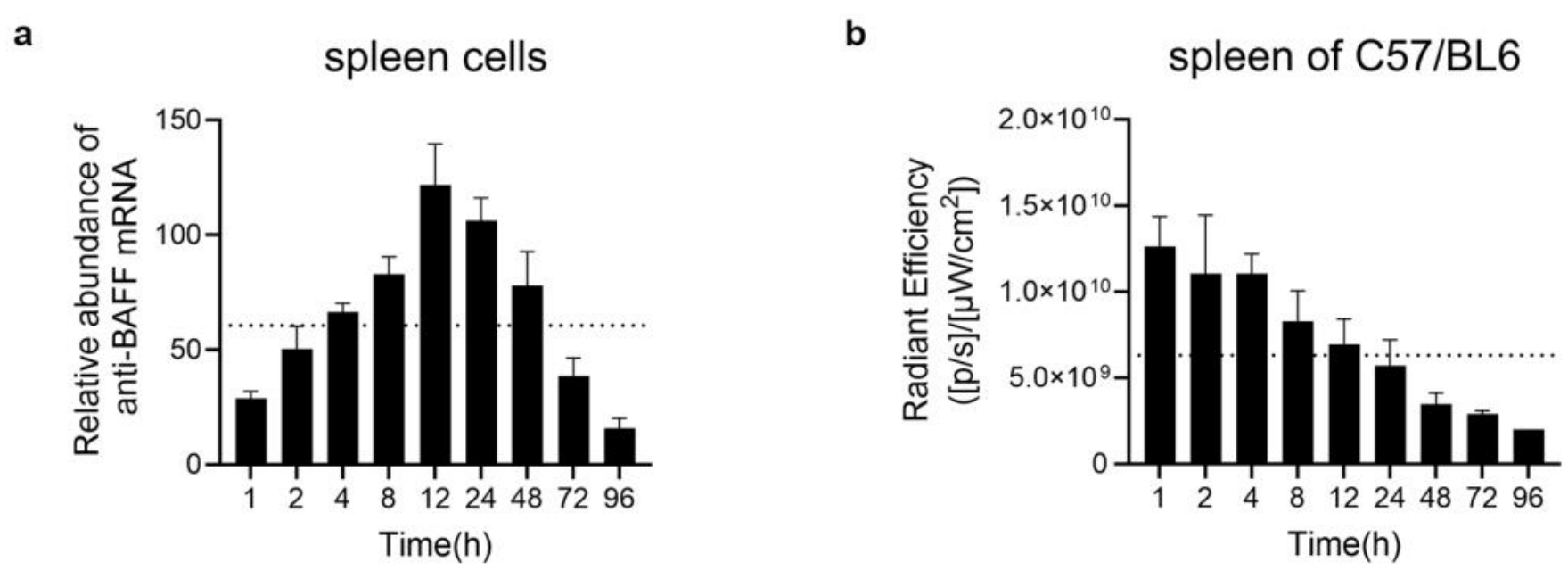

Figure S2 *In vivo* half-life of CD19@anti-BAFF^{mRNA} Exo.

(a) The relative abundance of anti-BAFF mRNA in the spleen was quantified by qPCR at 1, 2, 4, 8, 12, 24, 48, 72, 96 hours after the injection of CD19@anti-BAFF^{mRNA} Exo. The dashed line corresponds to 50% of the detected peak value for the relative abundance of mRNA levels. (b) The radiant efficiency of CD19@anti-BAFF^{mRNA} Exo (stained with DiR) in the spleen was statistically analyzed at 1, 2, 4, 8, 12, 24, 48, 72, 96 hours after injection. The dashed line corresponds to 50% of the detected peak value for the relative radiant efficiency.

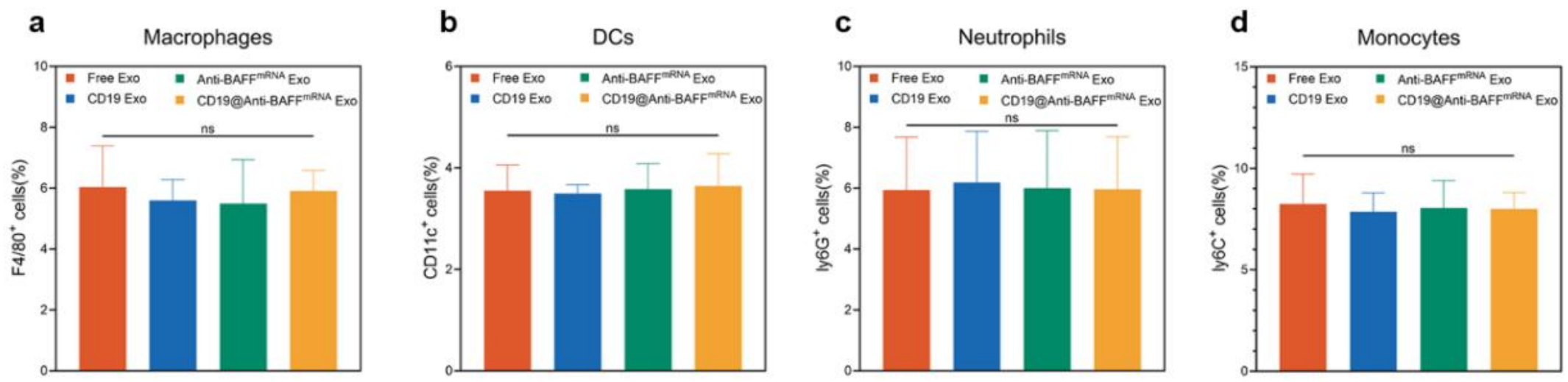

Figure S3 Proportions of immune cells in splenic cells of ALD-DNA induced lupus mice treated by different groups of exosomes.

(a) Proportions of macrophages, **(b)** DCs, **(c)** neutrophils and **(d)** monocytes in splenic cells of ALD-DNA induced lupus mice treated by different groups of exosomes. Data are presented as the mean±SEM of three biological replicates. ns: not significant.

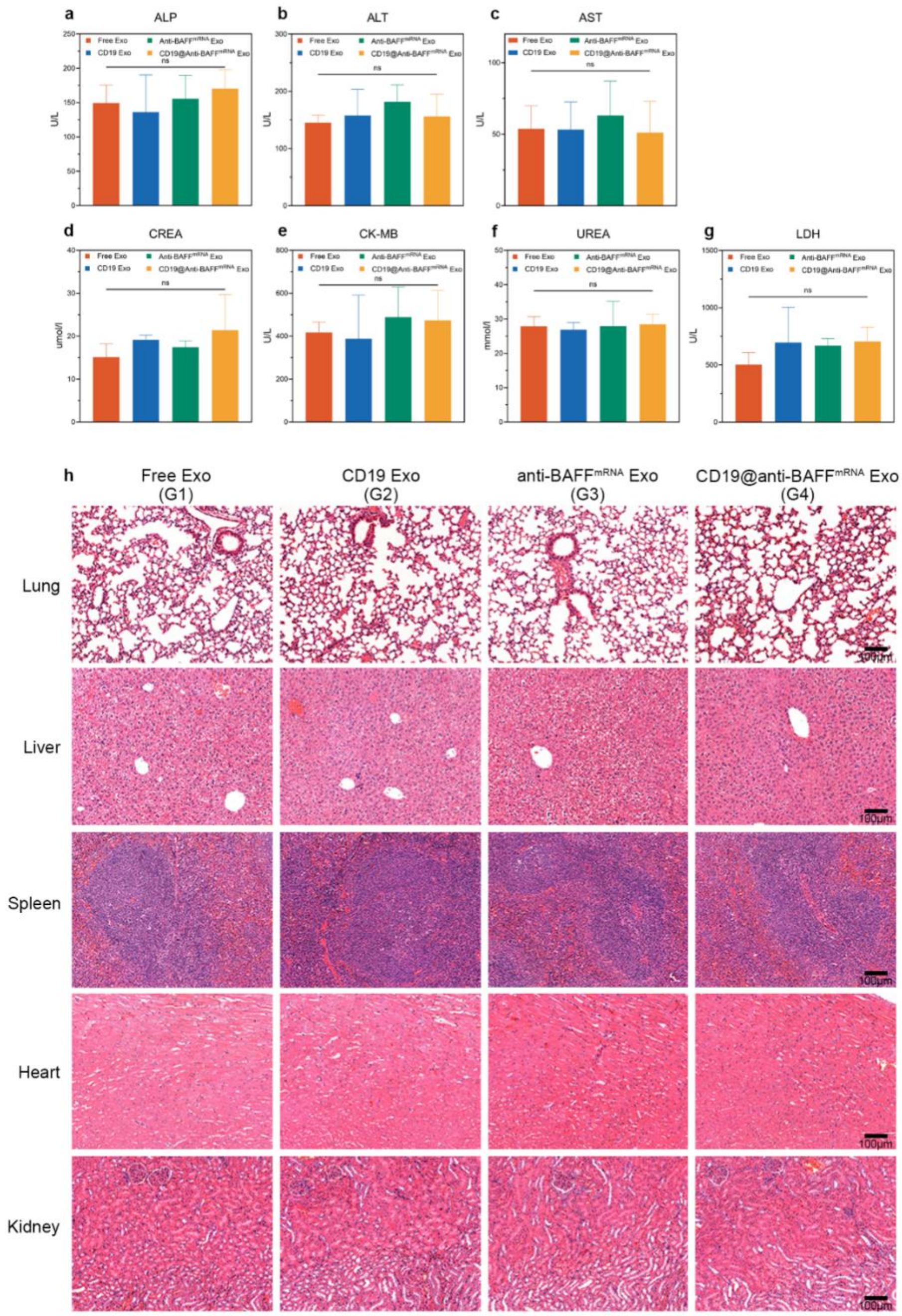

Figure S4 Biocompatibility of CD19@anti-BAFF^{mRNA} exosomes.

(a-g) The levels of biochemical indicators (ALP, ALT, AST, CREA, CK-MB, UREA and LDH) of wild-type mice treated with different groups of exosomes. **(h)** Representative images of H&E-stained slices of major organs (lung, liver, spleen, heart and kidney) of wild-type mice treated with different groups of exosomes.
